# Supplementary material for: Evaluating the impact and cost-effectiveness of typhoid conjugate vaccine schedules across diverse settings: A multi-model comparison
Source: PLoS Med. 2026 Oct 13;23(10):e1005280. doi: 10.1371/journal.pmed.1005280 (PMC13686463; doi:10.1371/journal.pmed.1005280)
Supplement: Supplementary file 1 [file pmed.1005280.s010.pdf]

Yale SCHOOL OF PUBLIC HEALTH

PO Box 208034
New Haven CT 06520-8034
publichealth.yale.edu

courier
60 College Street
New Haven CT 06510

June 29, 2026

Dear Dr. Cunha,

Thank you for the opportunity to revise our manuscript, "*Evaluating the Impact and Cost-Effectiveness of Typhoid Conjugate Vaccine Schedules Across Diverse Settings: A Multi-Model Comparison*", in response to your and the reviewers' feedback. We found the comments to be very helpful in ensuring the clarity of our analysis. Please find the reviewers' and editor's comments and our responses below.

We appreciate your time and consideration of this study for publication in *PLOS Medicine*.

Sincerely,

Catherine Wenger, MPH
(on behalf of the co-authors)
Department of Epidemiology of Microbial Diseases
Yale School of Public Health
catherine.wenger@yale.edu

<u>Response to reviewers:</u>

Reviewer Comment	Author Response
**Reviewer 1:** This study represents a significant technical contribution, harmonizing four transmission models (Burnet, IDM, Stanford, and Yale) to address critical policy questions regarding TCV waning. These findings are directly relevant to the WHO Strategic Advisory Group of Experts (SAGE), evaluating optimal schedules across varying incidence archetypes. Furthermore, the inclusion of healthcare-payer economic modeling and the focus on "fast" versus "slow" waning scenarios provide a robust framework for national decision-making.<br><br>As a member of the IVIR-AC who has reviewed the preliminary results, I find that the authors have addressed all previous comments and suggestions. Below are additional points for consideration.	We sincerely thank the reviewer for their previous review of this work and continued feedback in this review. We have addressed their specific comments below
**R1:** While the manuscript includes the recommended PSA, the resulting prediction intervals remain wide, particularly regarding DALYs averted and cost-effectiveness. In Figure 3, scenarios involving slow waning	We appreciate the challenge of interpreting these results and agree that more clearly providing the quantification of our certainty would make our results more useful for informing policymakers. The percentage of simulations in which a given schedule outperforms other schedules is indicated by the shading in Figures 3 and 4 and can be

with medium incidence and fast waning with high incidence show inconsistent results across models regarding the optimal age for routine vaccination (9 months vs. 2 years vs. 5 years). To assist policymakers in understanding the stability of these recommendations in contested epidemiological settings, please consider quantifying the percentage of simulations in which a 5-year routine schedule (or the recommended "optimal" schedules) outperforms a 9-month schedule (or other schedules) under fast-waning assumptions.	ascertained from the CEACs provided in the supplement (for which the y-axes show the probability that each strategy has the highest net benefit at a given willingness to pay). We have included the range of probabilities of highest net benefit across models throughout our results to illustrate the uncertainty in our findings.<br><br>For example:<br><br>*"For the slow-waning scenario, there is considerable uncertainty as to the optimal strategy, with routine vaccination at either 2 or 5 years of age preferred (probability of highest net benefit [PNB] = 15–50% and 7–42%, respectively), while for the fast-waning scenario, all models find that routine vaccination at 5 years is the preferred strategy at WTP thresholds >$1,250 per DALY averted (PNB = 25–55%) (see CEACs in Section 3 of S7 Text for PNB values)."* (lines 661-666, tracked version)
**R1:** The primary analysis identifies a 9-month routine dose plus a 5-year booster as the most optimal strategy. However, the current models assume that booster doses restore efficacy to the high levels of the primary dose. Given the lack of empirical evidence regarding TCV booster performance, the manuscript should explicitly discuss the impact of this assumption on the projected long-term effectiveness.	We agree that this is an important simplifying assumption to acknowledge. While immunogenicity data suggest that anti-Vi IgG levels are slightly higher following receipt of a booster dose compared to the primary dose, we made the simplifying assumption that VE was the same as following a primary dose at 5 years of age, since there is no efficacy data for booster doses and it is difficult to control for age in the immunogenicity studies. We added the following sentence to the limitations:<br><br>*"Booster doses were assumed to restore protection to a level equivalent to a primary dose received at 5 years of age. While immunogenicity data suggest there may be slightly higher levels of*

	anti-Vi IgG following receipt of a booster dose compared to primary doses, it is difficult to control for age in these analyses and there is no corresponding efficacy data. Therefore, we opted for a conservative assumption, potentially underestimating the long-term benefits of booster strategies." (lines 960-965)
**R1:** The Stanford model consistently predicts lower vaccine impact in very high-incidence settings compared to the IDM or Yale models. While the SI attributes this to a high force of infection leading to natural infection before vaccine eligibility, the discussion requires a more transparent comparison of structural assumptions. Specifically, please consider comparing Stanford's "hybrid mode of protection" against the compartmental structures used by Yale and Burnet, perhaps by presenting the mean age of first infection across all four models. This would provide necessary clarity on these divergent outcomes.	We agree that an additional analysis of the age of infection would better clarify the differences in vaccine impact we observe between models. All models were fit to the same age distribution of cases, and therefore, the average age of infection is similar between all models and incidence settings. Instead, we added a supplemental analysis showing the cumulative proportion of individuals ever infected by age group to illustrate that for the Stanford model, due to the high force of infection and fitted age-specific parameters, a much higher proportion of individuals are infected prior to 9 months of age compared to the other models, especially in the very high incidence setting.<br><br>We included the following figure to illustrate this, and reference it in the main text:<br><br>*"The predicted vaccine impact for the Stanford model was also substantially lower in very high incidence settings; this can be explained by a high estimated force of infection, such that individuals were first infected before being vaccine-eligible, thus reducing the benefit of vaccination (Figure 2.1 in S7 Text). The high force of infection combined with asymptomatic transmission from vaccinated individuals may also contribute to the*

	quicker rebound in cases observed in this setting." (lines 812-817)<br><br>In S7 Text:<br><br>*Figure 2.1 Median cumulative proportion ever infected by age group across models and incidence settings. The plotted values are the cumulative median proportion of individuals ever infected in each age group and 95% prediction interval across simulations. The Stanford results do not account for previously infected susceptible individuals, leading to an underestimation of the proportions (although this is likely small given the long duration of natural immunity estimated for this model).*<br><br>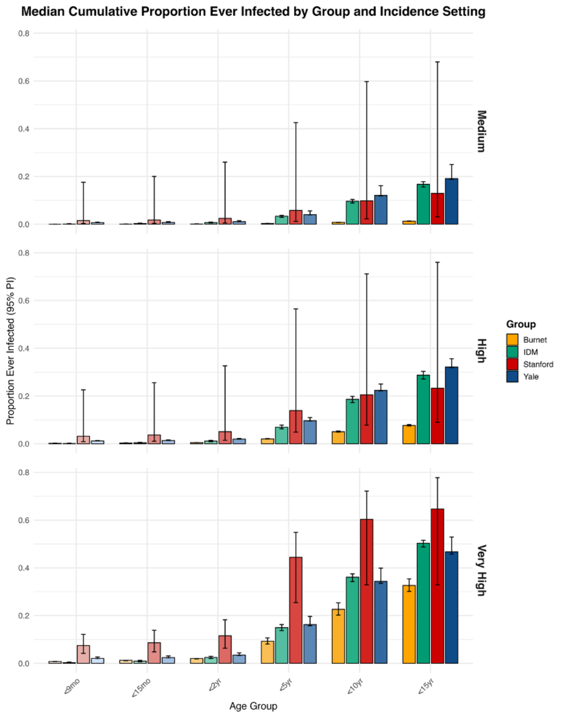

**Reviewer 2:** The study utilized 4 different models to draw overall conclusion on the impact and cost-effectiveness of TCV. The manuscript was well written and methods were clearly described. Meaningful insights for TCV vaccination strategies were provided across various settings with different incidence, CFR and VE waning rates. My main comments are related to what protection is referred to for TCV VE, and the differences in vaccine cost across strategies.	We thank the reviewer for their positive assessment and address their specific comments below.
**R2:** Please state clear what vaccine effectiveness of TCV refers to, e.g. protection against infection, disease or severe disease?	TCV vaccine effectiveness refers to protection against disease, i.e. blood-culture-confirmed typhoid fever. We have added clarifying text in Table 1 and in line 370:<br><br>*"...we estimated age-specific vaccine efficacy (VE) and duration of protection against disease (blood-culture-confirmed typhoid fever) for ages 9 months to <5 years and 5 to 15 years…"*
**R2:** Supplementary 5, VE against infection was used in the Burnet, IDM and Yale model, while VE for symptomatic disease was used in the Stanford model. There are likely differences between VE against infection and symptomatic disease. Could the authors give an overall description on how these have affected VE targets, waning and the main findings, and if any assumptions have been made?	The reviewer is correct that these structural assumptions create differences in the underlying dynamics of each model. Since there is a lack of data regarding TCV protection against infection, the Yale, IDM, and Burnet models make the simplifying assumption that protection against infection and protection against disease are equivalent, and they vary the relative infectiousness of chronic carriers to calibrate to the observed indirect protection from TCV observed in the Bangladesh cluster RCT. (This was explored in depth for the Yale model in a separate study:

	https://www.medrxiv.org/content/10.64898/2025.12.05.25341721v1.) Therefore, after calibration and validation, the difference between the use of VE against infection versus disease does not impact the initial observed outcomes, and all models successfully reproduce the clinical endpoints of the trials.<br><br>However, there may be differences in the herd immunity over time based on these different assumptions, which could potentially explain the quicker rebound in cases in the Stanford model compared to the other models in the slow waning setting (in fast waning settings, rebounds are likely more mediated by the duration of protection).<br><br>This has been clarified in the text here:<br><br>*"The models also have different mechanistic interpretations of the VE data, assuming vaccine protection against disease (Stanford) versus infection (Burnet, IDM, Yale); while this does not impact the initial observed outcomes of vaccination, it may contribute to the differences in calibrated effects as well as underlying disease dynamics post-vaccine introduction."* (lines 387-415)<br><br><br>*"The predicted vaccine impact for the Stanford model was also substantially lower in very high incidence settings; this can be explained by a high estimated force of infection, such that individuals were first infected before being vaccine-eligible, thus reducing the benefit of vaccination (Figure 2.1 in S7 Text). The high force of infection combined with asymptomatic transmission from vaccinated individuals may also contribute to the*

	quicker rebound in cases observed in this setting." (lines 812-817)
**R2:** Table 6.3.1, for the probability of outpatient treatment, please clarify "Compliment of P(inpatient treatment)"	This means 1 – the probability of inpatient treatment. (There was a typo; we meant "complement", not "compliment".)<br><br>The table has been updated for clarification:<br><br>*"1 – Probability of inpatient treatment"* (S6 Text Table 3.1)
**R2:** Table 2, the vaccine cost for routine vaccination at 9 months was $115-129 thousand per 100,000 population, reduced to $77-79 thousand per 100,000 population for routine vaccination at 5 years. What is the main reason for this remarkable 30-40% difference? Would the comparison between strategies significantly impacted by this drop in vaccine cost?	The main reason for this difference in cost is due to the target population and coverage estimates used for these different strategies. We assume lower coverage for routine vaccination at 5 years (based on school attendance), which translates to fewer doses. More importantly, we assume the catch-up campaign is only targeted to the 5-15 year age group, whereas for the "Routine vaccination at 9 months with a catchup campaign" strategy, we assume the campaign is targeted to children 9 months to 15 years. Therefore, many additional doses are administered for the 9-month strategy (Table 2.3 in S7 Text). We have clarified this when discussing the results in the text (lines 632-636).<br><br>This difference in costs may impact the preference for routine vaccination at older ages especially when waning is fast and negates the health benefits of more costly high-coverage campaigns, as reflected in the results of the cost-effectiveness analysis.
**R2:** Figure 1 shows that the trend of incidence was different for IDM, in particular with higher incidence than the no vaccine strategy after around 10 years.	We have updated our explanation for this in the Results section (lines 537-540):<br><br>*"...this is due to the complexity of calibrating agent-based models and the long pre-vaccination time horizon needed to reach a steady-state*

What is the main difference in the IDM model which explained the difference? Would the authors like to comment on this phenomenon?	*distribution of immunity in the simulated population."*
**Reviewer 3:** This article is on evaluating the cost effectiveness of different typhoid vaccine schedules across various settings. As a statistical reviewer, there are elements of the paper which other reviewers with primary experience in health economics and/or typhoid disease characteristics may be better placed to comment on. However, in my opinion the article is generally well written with clear aims, relevant methods used and interpretation of results that are reasonable. The article also benefits from having separate modelling done using different approaches to give some degree of sensitivity checking to results. There are though some aspects of the article which I don't think are fully addressed at present.	We appreciate the reviewer's assessment and have addressed the points raised below.
**R3:** With any modelling of this sort, irrespective of the choice of model the interpretation of results is heavily dependent on the assumptions that are being used. There does appear to have been substantial efforts made to address this as part of the article as far as is possible. For some assumptions though I think it is relevant to	We agree that this is an important takeaway message and expanded the discussion of the study's limitations related to the CFR:<br><br>*"Still, the overall CFR estimates were derived from a limited number of studies with a wide range of values, particularly in Africa, where the point estimate is <1% but the 95% CI extends up to 6.22%. This leads to greater decision*

emphasise in conclusions that there is a wide range of source data estimates (eg in Africa case fatality risk 95% CI 0.10% - 6.22%) that could potentially have a non-negligible impact on results. This is further illustrated in results where as probably anticipated the CFR value has a substantial impact on the outcomes (lines 475-478, figure S7.9) but that there is a lot of uncertainty in this value from the source data.	*uncertainty resulting from this highly influential parameter."* (lines 988-991)
**R3:** In a similar manner to comment above, single values of vaccine effectiveness are given in table 1 but the referenced papers have reasonably wide confidence intervals for these values, and isn't clear whether what is used are the best/worst/mid-case scenario and how that could affect results	We only provide the point estimates for the VE in Table 1 due to the large amount of information contained in this table. The corresponding 95% confidence intervals of the VE estimates are presented in Table 1 of the S3 Text. The models used the estimates differently to reproduce vaccine effects and end up with model-specific distributions of uncertainty (which are presented in Table 2 of S3 Text). The Yale and IDM groups used parameter estimates from a statistical model that was fitted to both the point estimates and variances of the observed VE (see p. 3-4 and Figure 1 of S3 Text), whereas the Stanford and Burnet groups fitted the "predicted VE" from their model to the point estimates of the observed VE (e.g. see Figure 2.3 and Figure 2.4 in the S5 Text).<br><br>*"This resulted in different modeled vaccine effects and uncertainty distributions that span the results of the trials."* (lines 386-387)
**R3:** Although acknowledged as a limitation in lines 622-627 and	We agree that WASH and other interventions are important components of addressing typhoid

that this may be considered as part of future analysis, it seems a substantial drawback that the vaccination is considered in isolation as to it's effectiveness/cost when there are potentially other sanitation or hygiene interventions that could instead be of equal or potentially even greater benefit than the vaccine. It may be difficult, if not impossible, to include this in models and alternative approaches are unlikely to be as relatively quick to apply as a vaccine would be. Nonetheless, I think would add substantially to the paper if more detail as to feasibility of other approaches (cost, time, cases and outcomes averted) could be outlined to place the vaccine approach in context.	burden. We attempted to put vaccination into context through the following addition to the discussion:<br><br>*"Vaccination alone will not eliminate typhoid, so TCV introduction should be considered in conjunction with water, sanitation, and hygiene (WASH) efforts and plans for antimicrobial stewardship. While the benefits of WASH interventions would extend well beyond their impact on typhoid, it has been estimated that over $1 billion would need to be invested over a 10-year period to achieve basic WASH infrastructure in a low-income country [36]. That level of investment is likely to be challenging. Vaccination serves as a cost-effective strategy to mitigate disease burden while longer-term WASH efforts are pursued."* (Lines 926-933)
**R3:** Catch-up campaigns are mentioned throughout but as far as I can tell it isn't explicitly stated what this involves and additional costs/times that may be relevant to consider. Additionally I don't think it is clear as to whether this is being stated as applicable for all methods to imply that higher vaccination of all eligible individuals would be achievable than what is specified as being assumed in table 1 (uniform 0.5-1 from measles vaccine).	We have included additional details about what we mean by catch-up campaigns on lines 255-257:<br><br>*"Each strategy included a catch-up campaign upon introduction, in which vaccines were administered to all individuals between the age of routine vaccination up to 15 years old over a four- to twelve-week period."*<br><br>While we assume different delivery costs for campaign doses (see Table 6.3.1 in the Supplement), we did not consider additional start-up costs for catch-up campaigns due to the limited data on campaigns conducted without Gavi support. We highlighted this as a limitation in lines 994-996:

	"Finally, we did not include start-up costs for the implementation of new TCV strategies, which may increase the WTP threshold at which vaccination is cost-effective."<br><br>Nevertheless, our estimates of the per-dose delivery costs of catch-up campaigns are in line with recent estimates for TCV (e.g. https://journals.plos.org/plosone/article?id=10.1371/journal.pone.0351148).<br><br>We assume all catch-up campaigns achieve a coverage level similar to measles campaign coverage (rather than applying a uniform multiplier) because campaigns are typically immunization events where the coverage achieved depends on a one-time mobilization of the health system to actively reach children. Furthermore, the few coverage estimates we do have for TCV campaigns are comparable to MCV. For routine immunization, we assume only a fraction of measles coverage or school attendance could be achieved for routine doses administered outside of the standard EPI schedule because it relies on passive attendance for vaccination, and we expect uptake to be lower than for a known vaccine administered at a well-established EPI visit. This additional explanation was added to the S4 Text.
**R3:** The models seem to assume that implementation will be possible at a fixed point in time (eg 9 months) with some catch up to complete coverage (as above comment). Although this may be all that is reasonable to include in terms of modelling, I think that some comment on the implementation of this is justified	Almost all of the 12 countries that have introduced TCV to date have done so by introducing the vaccine into routine vaccination programs (EPI schedule) at 9 or 15 months of age with a catch-up campaign to 15 years of age. Therefore, these scenarios reflect the reality of how TCVs are currently being implemented. Whether it is feasible to delay the first dose to 2 or 5 years of age, which are outside the typical EPI schedule, was discussed at length in both the SAGE typhoid

and what sort of visit window may be possible in practice as this would also affect whether single dose or booster strategies could be achievable.	working group and SAGE deliberations. Stakeholder interviews were conducted by the SAGE typhoid working group, and most respondents were not opposed to the idea of delaying the first dose to 2 years of age or older, although they recognized there would be trade-offs in terms of lower coverage. Ultimately, the WHO SAGE recommendation based on these findings is more permissive of the age-range for routine vaccination (9-24 months recommended in high and very high incidence settings and 2-5 years in medium incidence settings with a high CFR). We added the following sentence to the Discussion (lines 923-925):<br><br>*"While we modeled scenarios with routine vaccination at set ages, the updated WHO recommendation allows for more flexibility in terms of when TCV may be delivered [6]."*
**R3:** It isn't obvious as to why 97.5% confidence intervals are specified in lines 211-212 instead of the more usual 95% level and if this implies one sided tests.	This is how the clinical trial reported the confidence intervals for total and overall protection in Bangladesh (https://doi.org/10.1016/S0140-6736(21)01124-7). We did not calculate these estimates and only report the RCT results used for validating/calibrating the models.
**R3:** It is stated in lines 97-98 that the majority of disease burden is for children under 15 years old with a peak in this setting of between 2-4 year old. I don't believe though if this is what is then seen with model output in figure 1, in that the uniform distribution of non-vaccine group suggests incidence remains constant all the way between 0-20	Figure 1 is showing the overall incidence of typhoid fever in all age groups of the simulated population, in which births and deaths are occurring over time, for a period of 20 years after vaccine introduction. The age distributions of cases that are captured within this overall incidence are illustrated in S2 Text Figure 1 as well as in the S5 Text, where we show each model's fit to the age-specific incidence data. We have clarified the interpretation of figure 1 a/b in the caption.

years instead of also changing/reducing over time.	
**R3:** Some of the cells for Stanford are missing in supplementary table of model characteristics (S1_Table_model_characteristics.xlsx)	The missing information has been completed in S1_Table_model_characteristics.xlsx.
**Reviewer 4:** This manuscript aims to address an important question regarding the optimal schedule for typhoid vaccination given its waning protection in terms of economic and health impact using four different models. A few aspects could be refined.	We thank the reviewer for their comments and have addressed them below.
**R4:** Page 4, "In 2018, the World Health Organization (WHO) recommended the introduction of the typhoid conjugate vaccine (TCV) into the childhood routine immunization programs for infants and children aged 6 months to 15 years old in endemic countries, but vaccine adoption has been slow ": Can the authors clarify what the current WHO recommendation regarding TCV use or implementation strategies.	This is the most current recommendation prior to the updated recommendation that will come out of this analysis. We have clarified this in the introduction.<br><br>*"Since 2018, the World Health Organization (WHO) has recommended the introduction of the typhoid conjugate vaccine (TCV) into the childhood routine immunization programs for infants and children aged 6 months to 15 years old in endemic countries [5], but vaccine adoption has been slow. To date, 12 countries have introduced TCVs, with routine vaccination at 9 or 15 months of age and an initial catch-up campaign targeting children up to 15 years of age [6]."* (lines 167-170)
**R4:** Page 4, "Vaccine efficacy data from TCV trials in children less than 15 years in Malawi and	We agree that these details are important for setting up the rationale for re-evaluating the

Bangladesh show differences in the duration of vaccine protection": It would be helpful if the authors elaborate on observed differences between the two settings and what their implications for model assumptions?	recommendation for TCVs and have added the following sentences in that paragraph:<br><br>*"This suggests that booster doses may be needed in settings like Bangladesh in order to sustain protection against typhoid for the key at-risk age groups. Considering heterogeneous typhoid epidemiology, TCV waning, and increasingly constrained resources, which potentially limit childhood immunization efforts, re-assessing vaccine impact by modeling these different conditions can guide decision-makers to identify the optimal vaccine strategy for their local context."* (lines 197-202)
**R4:** Page 4, "Comparing the results of multiple models can inform the robustness of model predictions and indicate which assumptions and parameters have the greatest influence on the simulated impacts" : I think a concise overview of modeling approaches would provide a context given that they are repeatedly referred in methods and results.	We added the following details to the introduction to provide brief context for the modeling approaches before they are fully described in the methods section:<br><br>*"Comparing the results of multiple models that capture different complexities of typhoid natural history, such as environmental exposure and the role of chronic carriers, can inform the robustness of model predictions and indicate which assumptions and parameters have the greatest influence on the simulated impacts [15]."* (lines 206-210)<br><br>*"We compared two agent-based and two compartmental mathematical models…"* (line 234)
**R4:** Page 6, "Harmonized parameters and assumptions": what do the authors mean by harmonization?	Harmonized parameters and assumptions refers to components of the analysis for which all models use the same approach. We added the following clarification:<br><br>*"The models used harmonized input data and delivery scenarios to predict vaccine impact to*

	ensure a standardized and comparable baseline between the models while maintaining unique structural features and assumptions to allow for within- and between-model uncertainty." (lines 236-239)
**R4:** Page 16, "Figures 3 and 4 show the preferred vaccination strategies across WTP thresholds from $0 to $2,500 per DALY averted for the four models and the different incidence settings and waning scenarios [24] " : It is not clear what information is supported by reference 24. Please clarify. Consider in methods if it relates to methodological details.	This reference cites the guidance that informed our choice of willingness-to-pay range for figures 3 and 4. We have moved the reference to the methods section for clarity (now ref #25).<br><br>*"For the cost-effectiveness analysis, we used the net benefits framework to assess the optimal vaccine strategy from the health system perspective across a range of willingness-to-pay (WTP) thresholds from $0 to $2,500 (approximately equal to the average gross domestic product [GDP] per capita of a lower-middle-income country) [24, 25]."* (lines 477-481)
**R4:** Page 23, Can the authors clarify whether data from only two trials (Malawi / Bangladesh) were used or if any additional trials were considered as well, but not presented.	We only considered data from the Malawi and Bangladesh trials because their follow-up periods allow us to estimate the duration of protection of TCV in each setting. There is additional unpublished data from Nepal that suggests an intermediate duration of protection in this setting. We added the following clarification:<br><br>*"These trials were selected due to the extended duration of follow-up and divergent findings; estimates of VE were intermediate for a third trial conducted in Kathmandu, Nepal [23]."* (lines 376-378)

<u>Response to the Editor:</u>

Editor Comment	Author Response

We ask every co-author listed on the manuscript to fill in a contributing author statement, making sure to declare all competing interests.	Pending individual author response.
Please upload any figures associated with your paper as individual TIF or EPS files with 300dpi resolution at resubmission.	All figures included in this submission were evaluated by the NAAS tool and are now compliant with journal requirements.
We expect all researchers with submissions to PLOS in which author-generated code underpins the findings in the manuscript to make all author-generated code available without restrictions upon publication of the work. In cases where code is central to the manuscript, we require the code to be made available as a condition of publication. Authors are responsible for ensuring that the code is reusable and well documented. Please make any custom code available, either as part of your data deposition or as a supplementary file. Please add a sentence to your data availability statement regarding any code used in the study, e.g. "The code used in the analysis is available from Github [URL] and archived in Zenodo [DOI link]" Please review our guidelines at https://journals.plos.org/plosmedicine/s/materials-software-and-code-sharing and ensure that your	All code has been provided via stable DOI links here in our Data-Availability Statement:<br><br>*"All data produced in the present work are contained in the manuscript or available online for each model: Burnet Institute (https://doi.org/10.5281/zenodo.18687859), Institute for Disease Modeling (https://doi.org/10.5281/zenodo.20820053), Stanford University (https://doi.org/10.5281/zenodo.21011632), and Yale University (https://doi.org/10.5281/zenodo.18837535)."*

code is shared in a way that follows best practice and facilitates reproducibility and reuse. Because Github depositions can be readily changed or deleted, we encourage you to make a permanent DOI'd copy (e.g. in Zenodo) and provide the URL.	
Please ensure that the study is reported according to the most relevant guideline and include the completed corresponding checklist as Supporting Information. When completing the checklist, please use section and paragraph numbers, rather than page numbers. Please add the following statement, or similar, to the Methods: "This study is reported as per ... guideline (S1 Checklist)."	This study followed the CHEERS guideline. The CHEERS checklist was updated with more specific location references. A statement referencing the CHEERS checklist was added to the methods section (Lines 496-498).
Abstract: Please structure your abstract using the PLOS Medicine headings (Background, Methods and Findings, Conclusions). Please combine the Methods and Findings sections into one section.	The methods and findings are combined into one section in the abstract.
At this stage, we ask that you include a short, non-technical Author Summary of your research to make findings accessible to a wide audience that includes both scientists and non-scientists. The Author Summary should	An Author summary has been added after the abstract in the manuscript.

immediately follow the Abstract in your revised manuscript. This text is subject to editorial change and should be distinct from the scientific abstract. Ideally each sub-heading should contain 2-3 single sentence, concise bullet points containing the most salient points from your study. In the final bullet point of 'What Do These Findings Mean?', please include the main limitations of the study in non-technical language. Please see our author guidelines for more information: https://journals.plos.org/plosmedicine/s/revising-your-manuscript#loc-author-summary.	
Please express the main results with 95% CIs as well as p values. When reporting p values please report as p<0.001 and where higher as the exact p value p=0.002, for example. Throughout, suggest reporting statistical information as follows to improve clarity for the reader "22% (95% CI [13%,28%]; p</=)". Please be sure to define all numerical values at first use.	There are no p-values to report, as our methods do not include statistical tests. All confidence/prediction intervals have been updated to match the suggested reporting format.
Please include page numbers and line numbers in the manuscript file. Use continuous line numbers (do not restart the numbering on each page).	The manuscript file contains page numbers and continuous line numbers, per submission guidelines.

Please cite the reference numbers in square brackets. Citations should precede punctuation.	All citations are in square brackets and precede punctuation.
Please provide titles and legends for all figures and tables (including those in Supporting Information files).	All figures and tables have accompanying titles and legends.
Please define all abbreviations used in each figure/table (including those in Supporting Information files).	All abbreviations have been defined in the figure/table legends.
Please consider avoiding the use of red and green in order to make your figure more accessible to those with color blindness.	The authors acknowledge the importance of color choice, and all color sets were selected from palettes that are interpretable for all types of color blindness using the following website: https://davidmathlogic.com/colorblind/#%23D81B60-%231E88E5-%23FFC107-%23004D40
Please note that supplementary material will be posted as supplied by the authors. Therefore, please amend it according to the relevant comments outlined here: Please cite your Supporting Information as outlined here: https://journals.plos.org/plosmedicine/s/supporting-information	References to supplementary materials have been updated according to the provided journal guidance.<br>We have also split up the supplemental text into separate files for easier reference and reader navigation.
PLOS uses the numbered citation (citation-sequence) method and first six authors, et al.	The reference list follows the citation sequence and includes first six authors, et al. when applicable.
Please ensure that journal name abbreviations match those found	The journal names have been corrected in the citation list according to the NCBI database.

in the National Center for Biotechnology Information (NCBI) databases (http://www.ncbi.nlm.nih.gov/nlmcatalog/journals), and are appropriately formatted and capitalised.	
Where website addresses are cited, please include the complete URL and specify the date of access (e.g. [accessed: 12/06/2023]).	Access dates have been added to all website references.
If pertinent, please provide a diagram that shows the model structure, including how the natural history of the disease is represented, the process and determinants of disease acquisition, and how the putative intervention could affect the system.<br>Please provide a complete list of model parameters, including clear and precise descriptions of the meaning of each parameter, together with the values or ranges for each, with justification or the primary source cited and important caveats about the use of these values noted.<br>Please provide a clear statement about how the model was fitted to the data, including goodness-of-fit measure, the numerical algorithm used, which parameter varied, constraints imposed on parameter values, and starting conditions.	All of the following points are included in the manuscript or supplemental files.

For uncertainty analyses, please state the sources of uncertainties quantified and not quantified [can include parameter, data, and model structure].<br>Please provide sensitivity analyses to identify which parameter values are most important in the model.<br>Uncertainty estimates seek to derive a range of credible results on the basis of an exploration of the range of reasonable parameter values. The choice of method should be presented and justified.<br>Please discuss the scientific rationale for the choice of model structure and identify points where this choice could influence conclusions drawn. Please also describe the strength of the scientific basis underlying the key model assumptions.	
For studies that develop a prediction model or evaluate its performance, please ensure that the study is reported according to the TRIPOD statement (https://www.equator-network.org/reporting-guidelines/tripod-statement) and include the completed checklist as Supporting Information. Please add the following statement, or similar, to the Methods: "This study is reported as per the Transparent Reporting of a Multivariable Prediction Model for Individual	This study does not fall in the category of studies that use TRIPOD statements.

Prognosis Or Diagnosis (TRIPOD) statement (S1 Checklist)." For studies using machine learning, please use the TRIPOD-AI checklist. When completing the checklist, please use section and paragraph numbers, rather than page numbers.	
Please ensure that the study is reported according to the CHEERS guideline (available from: https://www.equator-network.org/reporting-guidelines/cheers) and include the completed checklist as Supporting Information. Please add the following statement, or similar, to the Methods: "This study is reported as per the Strengthening the Consolidated Health Economic Evaluation Reporting Standards 2022 (CHEERS 2022) Statement (S1 Checklist)." When completing the checklist, please use section and paragraph numbers, rather than page numbers.	The CHEERS checklist was updated with more specific location references. A statement referencing the CHEERS checklist was added to the methods section (Lines 496-498).
Please ensure that the funders and grant numbers match between the Financial Disclosure field and the Funding Information tab in your submission form. Note that the funders must be provided in the same order in both places as well. Currently, "Gates Foundation, Stanford King Center on Global	The funding information tab has been updated to match the funders listed in the financial disclosure statement.

Development,Stanford Propel program" are missing from the Funding Information	
Please ensure that the paper adheres to the PLOS Data Availability Policy (see http://journals.plos.org/plosmedicine/s/data-availability), which requires that all data underlying the study's findings be provided in a repository or as Supporting Information. For data residing with a third party, authors are required to provide instructions with contact information (web or email address) for obtaining the data. Please note that a study author cannot be the contact person for the data. PLOS journals do not allow statements supported by "data not shown" or "unpublished results." For such statements, authors must provide supporting data or cite public sources that include it.<br><br>We noticed that you used "unpublished data" in the manuscript. We do not allow these references, as the PLOS data access policy requires that all data be either published with the manuscript or made available in a publicly accessible database. Please amend the supplementary	The references to unpublished data have been removed from the submission. All other components of the paper have been checked to ensure compliance with PLOS's Data Availability Policy.

material to include the referenced data or remove the references.	
