# Supplementary material for: Evaluating the impact and cost-effectiveness of typhoid conjugate vaccine schedules across diverse settings: A multi-model comparison
Source: PLoS Med. 2026 Oct 13;23(10):e1005280. doi: 10.1371/journal.pmed.1005280 (PMC13686463; doi:10.1371/journal.pmed.1005280)
Supplement: Supplementary file 2 [file pmed.1005280.s011.pdf]

Response to the Editor:

Editor Comment	Author Response
* At this stage, we ask that you include a short, non-technical Author Summary of your research to make findings accessible to a wide audience that includes both scientists and non-scientists. The Author Summary should immediately follow the Abstract in your revised manuscript. This text is subject to editorial change and should be distinct from the scientific abstract. Ideally each sub-heading should contain 2-3 single sentence, concise bullet points containing the most salient points from your study. In the final bullet point of 'What Do These Findings Mean?' Please include the main limitations of the study in non-technical language.<br><br>Please see our author guidelines for more information: https://journals.plos.org/plosmedicine/s/revising-your-manuscript#loc-author-summary.	The second section of the author summary was reduced from four bullet points to three. Otherwise, the Author Summary follows PLOS Medicine guidelines.
* Please confirm that your title complies with PLOS Medicine's style. Your title must be nondeclarative and not a question. It should begin with main concept if possible. "Effect of" should be used only if causality can be inferred, i.e., for an RCT. Please place the study design ("A randomized controlled trial," "A	We confirm that our title follows the PLOS Medicine style guidelines.

retrospective study," "A modelling study," etc.) in the subtitle (ie, after a colon).	
* Please confirm that your abstract complies with our requirements, including format (three sections: Background, Methods and Findings, and Conclusions) and providing all the information relevant to this study type https://journals.plos.org/plosmedicine/s/submission-guidelines#loc-abstract	We confirm that our abstract complies with the PLOS Medicine requirements.
* Please ensure that the Introduction ends with a clear description of the study question or hypothesis.	Our introduction clearly states the study question we are trying to answer: identifying the optimal typhoid vaccination strategy under different incidence, waning, and economic scenarios. (lines 179-183)
* Please ensure that all abbreviations are defined at first use throughout the text.	The manuscript and all supplemental materials were reviewed, and all abbreviations are defined at first use in the text.
* Please confirm that all numbers presented in the abstract are present and identical to numbers presented in the main manuscript text.	The abstract has been reviewed and all numbers are now consistent with the results presented in the main manuscript text.

* Please include a statement regarding use or non-use of AI tools and technologies at the end of the Methods. Please specify the name(s) and version(s) of any tool(s) used and a description of their use, including how the AI-assisted or generated content was evaluated by authors, and a clear statement of which aspects of the study, article contents, data, or supporting files were affected or generated by AI tool usage. Please also include a statement specifying who retains responsibility for the final manuscript. See our guidelines for more information: https://journals.plos.org/plosmedicine/s/ethical-publishing-practice#loc-artificial-intelligence-tools-and-technologies.	The following section has been added to the manuscript:<br><br>**Use of artificial intelligence**<br><br>ChatGPT (OpenAI; GPT-5.5) and Claude (Anthropic; Sonnet Versions 2-4) were used solely as a technical aid to assist with coding tasks including debugging and troubleshooting code-related issues, code streamlining and parallelization, extensions' structuring, review, and repository preparation for public release. All scripts and outputs were reviewed and verified by the authors, who retained full responsibility for the study design, analyses, interpretation, final manuscript, and supporting materials.
* Please review your text for claims of novelty or primacy (e.g. 'for the first time') and remove this language. In addition, please check that any use of statistical terms (such as trend or significant) are supported by the data, and if not please remove them.	No references to the novelty or primacy of our methods were made in this manuscript.

* In the last sentence of the Abstract Methods and Findings section, please describe the main limitation(s) of the study's methodology.	A sentence has been added to the abstract describing the study's limitations.<br>*"The study findings are limited by the data available to inform indirect vaccine protection and other simplifying assumptions made in this analysis."*
* In the abstract, please include the important dependent variables that are adjusted for in the analyses.	Not applicable.
* In the author summary, in the final bullet point of 'What Do These Findings Mean?', please include the main limitations of the study in non-technical language.	A line describing the study limitations was added to the last section of the Author Summary.<br>*"The limitations of this study include the sparse data available to inform indirect vaccine protection and other simplifying assumptions made in this analysis."*
FUNDING STATEMENT<br><br>* The funding statement should include: specific grant numbers, initials of authors who received each award, URLs to sponsors' websites. Also, please state whether any sponsors or funders (other than the named authors) played any role in study design, data collection and	To clarify the authors' roles in this study as employees of one of the funders, the following updates have been made to the funding statement:<br>"**Funding:** The Burnet Institute (AM, NS, RA), Stanford University (TM, NCL, JA), and Yale University (CW, ZP, VP) groups received support for this work from the World Health Organization (2024/1528504-0 to VEP and RGA)

analysis, the decision to publish, or preparation of the manuscript. If they had no role in the research, include this sentence: “The funders had no role in study design, data collection and analysis, decision to publish, or preparation of the manuscript.”<br><br>* It appears that one or more study authors is affiliated with one or more of the agencies that funded the study. Thus, the statement “The funders had no role in study design, data collection and analysis, decision to publish, or preparation of the manuscript” does not apply. Please revise the Financial Disclosure accordingly, as in "[Author name] is [author's role] at [funding agency]. The funders had no other role in study design…..”	(https://www.who.int/) and the Gates Foundation through the Vaccine Impact Modelling Consortium (INV-034281 to VEP and NS) (https://www.vaccineimpact.org/). The Institute for Disease Modeling (KHG, AK, JG), doing this work as a research group within the Gates Foundation, was solely funded by the Gates Foundation (https://www.gatesfoundation.org/). JRA and NCL report additional funding support from the Gates Foundation and Stanford King Center on Global Development. TM reports additional funding support from the Stanford Propel program. The World Health Organization advised on the study design and provided feedback on the analysis through the Strategic Advisory Group of Experts typhoid working group and the Immunization and Vaccine-related Implementation Research Advisory Committee. **JG is a Senior Research Manager, KHG is a Research Scientist, and AK is a Senior Research Scientist at the Gates Foundation. These authors are not involved in the allocation of funds from the Gates Foundation, and served solely as researchers on this project. The funders played no other role in the study design, data collection and analysis, decision to publish, or preparation of the manuscript.**”

COMPETING INTERESTS STATEMENT<br><br>* All authors must declare their relevant competing interests per the PLOS policy, which can be seen here: https://journals.plos.org/plosmedicine/s/competing-interests For authors with ties to industry, please indicate whether any of the interests has a financial stake in the results of the current study.	All authors have confirmed the details in the current competing interests statement.
* Please provide titles and legends for all figures and tables (including those in Supporting Information files). Please define all acronyms used in each figure or table in its corresponding legend.	The manuscript and all supplemental materials were reviewed, and all figures and tables have titles and legends. All abbreviations are defined in the corresponding legends.
* Please consider avoiding the use of red and green in order to make your figure more accessible	The authors acknowledge the importance of color choice, and all color sets were selected from palettes that are interpretable for all types of color blindness using the following website: https://davidmathlogic.com/colorblind/#%23D81B60-%231E88E5-%23FFC107-%23004D40<br>All color palettes were tested for CVD accessibility.
* Please provide the unadjusted comparisons as well as the adjusted comparisons in all relevant Tables	Not applicable.
* Please specify the variables controlled for in all relevant Tables	Not applicable.

ACKNOWLEDGMENTS<br><br>* Please include an Acknowledgments section in your manuscript. The Acknowledgements should not include funder information.	“We would like to acknowledge the World Health Organization Strategic Advisory Group of Experts Working Group on typhoid immunization and the Immunization and Vaccines Related Implementation Research Advisory Committee for their technical guidance and feedback throughout this project. We would also like to thank members of the TyVAC consortium for generating data used in this study.”
MODELING STUDIES<br><br>For more details on the requests below please see Geoffrey P Garnett, Simon Cousens, Timothy B Hallett, Richard Steketee, Neff Walker. Mathematical models in the evaluation of health programmes. (2011) Lancet DOI:10.1016/S0140-6736(10)61505-X.<br><br>* Please provide a diagram that shows the model structure, including how the disease natural history is represented, the process and determinants of disease acquisition, and how the putative intervention could affect the system.	All model diagrams and descriptions of the model structures can be found in S5 Text, particularly in figures 2.1, 3.1, 4.1, and 5.1.
* Please provide a complete list of model parameters, including clear and precise descriptions of the meaning of each parameter, together with the values or ranges for each, with justification or the primary	All model parameters are listed and described in the descriptive text for each model in S5 Text, the S5 Text tables 2.1, 3.1, 4.1, and 5.1, and S1 Table.

source cited, and important caveats about the use of these values noted.	
* Please provide a clear statement about how the model was fitted to the data including where relevant goodness-of-fit measure, the numerical algorithm used, which parameter varied, constraints imposed on parameter values, and starting conditions.	The model fitting processes are described in S5 Text in sections 2-5.
* For uncertainty analyses, please state the sources of uncertainties quantified and not quantified this can include parameter, data, and model structure.	Uncertainty analyses are described fully in the methods section and S2-6 texts. These sources of parameter and data uncertainty are summarized in Table 1 of the manuscript. Model structure uncertainty is inherently evaluated via the comparison of 4 different model structures, described in lines 219-228. Lines 327-337 describe how this uncertainty is reflected in the model results.
* Please provide sensitivity analyses to identify which parameter values are most important in the model. Uncertainty estimates seek to derive a range of credible results on the basis of an exploration of the range of reasonable parameter values. The choice of method should be presented and justified.	Methods relating to uncertainty analyses are described in lines 338-352 in the main manuscript. Sensitivity analysis results are described in lines 532-541 in the main manuscript and in Figures 4.1-4, 5.1-4, 6.1-4, and 9.1-2 in S7 Text.
* Please discuss the scientific rationale for this choice of model structure and identify points where this choice could influence conclusions drawn. Please also describe the strength of the scientific	All structural model assumptions are described in S5 Text and S1 Table. The impact these assumptions have on the results is discussed in lines 557-576 of the manuscript.

basis underlying the key model assumptions.	
