# Supplementary material for: Evaluating the impact and cost-effectiveness of typhoid conjugate vaccine schedules across diverse settings: A multi-model comparison
Source: PLoS Med. 2026 Oct 13;23(10):e1005280. doi: 10.1371/journal.pmed.1005280 (PMC13686463; doi:10.1371/journal.pmed.1005280)
Supplement: Supplementary file 4 [file pmed.1005280.s003.docx]

***S1 Supplementary Text: Demographic Data***

**Evaluating the Impact and Cost-Effectiveness of Typhoid Conjugate Vaccine Schedules Across Diverse Settings: A Multi-Model Comparison**

*Authors:*

Catherine G.C. Wenger^1,&^, Kyra H. Grantz^2,&^, Tigist F. Menkir^3,&^, Alina M. Muellenmeister^4,&^, Zeaan Pithawala^1^, Raymond Hutubessy^5^, Vittal Mogasale^5^, Alicia N.M. Kraay^2^, Nick Scott^4^, Romesh G. Abeysuriya^4,$^, Jason R. Andrews^3,$^, Jillian Gauld^2,$^, Nathan C. Lo^3,$^, Virginia E. Pitzer^1,$^

*Affiliations:*

1 Department of Epidemiology of Microbial Diseases, Yale School of Public Health, Yale University, New Haven, Connecticut, United States of America

2 Institute for Disease Modeling, Gates Foundation, Seattle, Washington, United States of America

3 Division of Infectious Diseases and Geographic Medicine, Department of Medicine, Stanford University, Stanford, California, United States of America

4 Burnet Institute, Melbourne, Victoria, Australia

5 Department of Performance, Finance and Delivery, World Health Organization, Geneva, Switzerland

^&^co-first authors

^$^co-last authors

Disclaimer: RH and VM are staff members of the World Health Organization. The views expressed are their personal ones and do not necessarily represent the views of their organization. KHG, AK, and JG are employees of the Gates Foundation; however, this study does not necessarily represent the views of the Gates Foundation.

# A. Demographic data

We harmonized the four epidemiological models by assuming the same population dynamics based on the demographic parameters of low- and lower-middle-income countries from the United Nations World Population Prospects [1]. We used crude birth rates and the proportion of the population by age group to fit age-specific all-cause death rates that result in a stable population size and age distribution over time. The crude birthrate was 22.8 births per 1000 persons per year, and the life expectancy was 68.88 years. The age composition of the population is detailed in Table A.1.

**Table A.1: Proportion of the population in each age group.**

| **Age group** | **Proportion of the population** |
| --- | --- |
| 0-<2 years | 0.044 |
| 2-<5 years | 0.064 |
| 5-<10 years | 0.105 |
| 10-<15 years | 0.102 |
| 15-<20 years | 0.096 |
| 20-<25 years | 0.089 |
| 25+ years | 0.502 |

**References**

1. World Population Prospects 2024 | Population Division [Internet]. United Nations; 2024 [cited 2025 Dec 11]. Available from: https://www.un.org/development/desa/pd/world-population-prospects-2024
