# Supplementary material for: Evaluating the impact and cost-effectiveness of typhoid conjugate vaccine schedules across diverse settings: A multi-model comparison
Source: PLoS Med. 2026 Oct 13;23(10):e1005280. doi: 10.1371/journal.pmed.1005280 (PMC13686463; doi:10.1371/journal.pmed.1005280)
Supplement: Supplementary file 5 [file pmed.1005280.s004.docx]

***S2 Supplementary Text: Incidence Data***

**Evaluating the Impact and Cost-Effectiveness of Typhoid Conjugate Vaccine Schedules Across Diverse Settings: A Multi-Model Comparison**

*Authors:*

Catherine G.C. Wenger^1,&^, Kyra H. Grantz^2,&^, Tigist F. Menkir^3,&^, Alina M. Muellenmeister^4,&^, Zeaan Pithawala^1^, Raymond Hutubessy^5^, Vittal Mogasale^5^, Alicia N.M. Kraay^2^, Nick Scott^4^, Romesh G. Abeysuriya^4,$^, Jason R. Andrews^3,$^, Jillian Gauld^2,$^, Nathan C. Lo^3,$^, Virginia E. Pitzer^1,$^

*Affiliations:*

1 Department of Epidemiology of Microbial Diseases, Yale School of Public Health, Yale University, New Haven, Connecticut, United States of America

2 Institute for Disease Modeling, Gates Foundation, Seattle, Washington, United States of America

3 Division of Infectious Diseases and Geographic Medicine, Department of Medicine, Stanford University, Stanford, California, United States of America

4 Burnet Institute, Melbourne, Victoria, Australia

5 Department of Performance, Finance and Delivery, World Health Organization, Geneva, Switzerland

^&^co-first authors

^$^co-last authors

Disclaimer: RH and VM are staff members of the World Health Organization. The views expressed are their personal ones and do not necessarily represent the views of their organization. KHG, AK, and JG are employees of the Gates Foundation; however, this study does not necessarily represent the views of the Gates Foundation.

# A. Incidence Data

To define the average incidence and age distribution of cases for each incidence archetype, we conducted a literature search to identify studies that conducted population-based or hybrid surveillance of typhoid fever. We did not include studies from low incidence settings (<10 cases per 100,000 person-years) as these are typically from high- or upper-middle-income countries in which typhoid is not endemic. Only studies that reported incidence rates for all age categories (0-<5, 5-<15, and 15+ years old) were included. We extracted data on blood-culture-confirmed case counts and person-time of follow-up, which were adjusted for the probability of seeking care, the proportion of cases meeting the case definition with blood drawn for culture, and blood culture sensitivity, as reported by the studies. The data extracted for each country was categorized into medium incidence (10-99 cases per 100,000 person-years), high incidence (100-499 cases per 100,000 person-years), and very high incidence (500+cases per 100,000 person-years) based on the adjusted overall incidence. We ultimately included 36 studies from 18 countries (Table A.1).

To achieve a finer breakdown of age groups, we used a subset of studies that reported cases and person-time separately for the 0-<2-year, 2-<5-year, 5-<10-year, and 10-<15-year age categories (but may not have reported all age groups) to calculate the ratio of incidence in the 0-<2-year age group compared to the 2-<5-year age group and the 5-<10-year age group compared to the 10-<15-year age group for each incidence setting. These ratios were then used to redistribute the cases in the 0-<5-year and 5-<15-year age groups of the complete data set into the finer age categories. From there, the person-time was divided based on the proportion of the population in each of the smaller age groups. We added up the cases and person-time in each age group across all of the studies in each of the three incidence settings, and age-specific incidence was calculated for the new age groups.

**Table A.1: Age distribution of incidence in each incidence archetype.**

| **Age groups (years)** | **Cases** | **Person-years (PY)** | **Incidence per 100K PY** |
| --- | --- | --- | --- |
| ***Medium incidence settings*** | | | |
| 0-<2y | 3 | 13801 | 22 |
| 2-<5y | 10 | 20074 | 50 |
| 5-<10y | 30 | 45891 | 66 |
| 10-<15y | 32 | 44580 | 71 |
| 15+y | 18 | 52249 | 34 |
| All | 93 | 176595 | 53 |
| ***High incidence settings*** | | | |
| 0-<2y | 39 | 24601 | 157 |
| 2-<5y | 185 | 35784 | 518 |
| 5-<10y | 275 | 71742 | 383 |
| 10-<15y | 235 | 69692 | 337 |
| 15+y | 450 | 350883 | 128 |
| All | 1184 | 552703 | 214 |
| ***Very high incidence settings*** | | | |
| 0-<2y | 53 | 3976 | 1343 |
| 2-<5y | 241 | 5784 | 4159 |
| 5-<10y | 359 | 13019 | 2757 |
| 10-<15y | 239 | 12647 | 1890 |
| 15+y | 367 | 64928 | 565 |
| All | 1259 | 100355 | 1255 |

**Figure A.1: Age distribution of incidence in each incidence archetype.** Incidence rates are adjusted for the probability of seeking care for febrile illness, the fraction of cases meeting the case definition for which blood was drawn, and the sensitivity of blood culture for typhoid diagnosis.

**Table A.2: Studies used to inform the age-specific incidence in archetypal settings.** Location, median year, and source of data is noted. Studies are categorized by the overall adjusted incidence of typhoid fever (medium: 10-99 cases per 100,000 person-years; high: 100-499 cases per 100,000 person-years; very high: 500+ cases per 100,000 person-years).

| **Location** | **Year of data collection** | **Source** |  |
| --- | --- | --- | --- |
| ***Medium incidence*** | | | |
| Bandim, Guinea-Bissau | 2012 | [1] |  |
| Butajira, Ethiopia | 2013 | [1] |  |
| Ibadan, Nigeria | 2018 | [2] |  |
| Isotry, Madagascar | 2012 | [1] |  |
| Moshi, Tanzania (rural) | 2013 | [1] |  |
| Imerinsiatosika, Madagascar | 2012 | [1] |  |
| Pikine, Senegal | 2012 | [1] |  |
| ***High incidence*** | | | |
| Blantyre, Malawi | 2016 | [3] |  |
| Agogo, Ghana | 2017 | [2] |  |
| Dhulikhel, Nepal | 2018 | [4] |  |
| East Champaran, India | 2019 | [5] |  |
| Nioko, Burkina Faso | 2012 | [1] |  |
| Karachi, Pakistan | 2018 | [4] |  |
| Karimganj, India | 2019 | [5] |  |
| Kathmandu, Nepal | 2018 | [4] |  |
| Kavuaya and Nkandu, Democratic Republic of the Congo | 2019 | [2] |  |
| Kolkata, India | 2004 | [6] |  |
| Kullu, India | 2019 | [5] |  |
| Lwak, Kenya | 2008 | [7] |  |
| Moshi, Tanzania (urban) | 2013 | [1] |  |
| Nandurbar, India | 2019 | [5] |  |
| Kibera, Kenya | 2013 | [1] |  |
| Polesgo, Burkina Faso | 2012 | [1] |  |
| North Jakarta, Indonesia | 2002 | [8] |  |
| Pemba, Tanzania | 2009 | [9] |  |
| Samarkand City, Uzbekestan | 2002 | [10] |  |
| ***Very high incidence*** | | | |
| Anantpur, India | 2019 | [5] |  |
| Chandigarh, India | 2019 | [5] |  |
| Communes Dong Thap, Vietnam | 1996 | [11] |  |
| Delhi, India | 1996 | [12] |  |
| Dhaka, Bangladesh | 2001 | [13] |  |
| Dhaka, Bangladesh | 2016 | [3] |  |
| Kathmandu, Nepal | 2016 | [3] |  |
| Kibera, Kenya | 2008 | [7] |  |
| Kolkata, India | 2005 | [6] |  |
| Sumatra, Indonesia | 1988 | [14] |  |

**References**

1. Marks F, von Kalckreuth V, Aaby P, Adu-Sarkodie Y, El Tayeb MA, Ali M, et al. Incidence of invasive salmonella disease in sub-Saharan Africa: A multicentre population-based surveillance study. The Lancet Global Health. 2017 Mar;5(3). doi:10.1016/s2214-109x(17)30022-0
2. Marks F, Im J, Park SE, Pak GD, Jeon HJ, Wandji Nana LR, et al. Incidence of typhoid fever in Burkina Faso, Democratic Republic of the Congo, Ethiopia, Ghana, Madagascar, and Nigeria (the severe typhoid in Africa programme): A population-based study. The Lancet Global Health. 2024 Apr;12(4). doi:10.1016/s2214-109x(24)00007-x
3. Meiring JE, Shakya M, Khanam F, Voysey M, Phillips MT, Tonks S, et al. Burden of enteric fever at three urban sites in Africa and Asia: A Multicentre population-based study. The Lancet Global Health. 2021 Dec;9(12). doi:10.1016/s2214-109x(21)00370-3
4. Garrett DO, Longley AT, Aiemjoy K, Yousafzai MT, Hemlock C, Yu AT, et al. Incidence of typhoid and paratyphoid fever in Bangladesh, Nepal, and Pakistan: Results of the surveillance for Enteric Fever in Asia Project. The Lancet Global Health. 2022 Jul;10(7). doi:10.1016/s2214-109x(22)00119-x
5. John J, Bavdekar A, Rongsen-Chandola T, Dutta S, Gupta M, Kanungo S, et al. Burden of typhoid and paratyphoid fever in India. New England Journal of Medicine. 2023 Apr 20;388(16):1491–500. doi:10.1056/nejmoa2209449
6. Sur D, von Seidlein L, Manna B, Dutta S, Deb AK, Sarkar BL, et al. The malaria and typhoid fever burden in the slums of Kolkata, India: Data from a prospective community-based study. Transactions of the Royal Society of Tropical Medicine and Hygiene. 2006 Aug;100(8):725–33. doi:10.1016/j.trstmh.2005.10.019
7. Breiman RF, Cosmas L, Njuguna H, Audi A, Olack B, Ochieng JB, et al. Population-based incidence of typhoid fever in an urban informal settlement and a rural area in Kenya: Implications for typhoid vaccine use in Africa. PLoS ONE. 2012 Jan 19;7(1). doi:10.1371/journal.pone.0029119
8. Punjabi NH, Agtini MD, Ochiai RL, Simanjuntak CH, Lesmana M, Subekti D, et al. Enteric fever burden in North Jakarta, Indonesia: A prospective, community-based study. The Journal of Infection in Developing Countries. 2013 Nov 15;7(11):781–7. doi:10.3855/jidc.2629
9. Thriemer K, Ley B, Ame S, von Seidlein L, Pak GD, Chang NY, et al. The burden of invasive bacterial infections in Pemba, Zanzibar. PLoS ONE. 2012 Feb 17;7(2). doi:10.1371/journal.pone.0030350
10. Srikantiah P, Vafokulov S, Luby SP, Ishmail T, Earhart K, Khodjaev N, et al. Epidemiology and risk factors for endemic typhoid fever in Uzbekistan. Tropical Medicine &amp; International Health. 2007 Jun 25;12(7):838–47. doi:10.1111/j.1365-3156.2007.01853.x
11. Lin FY, Vo AH, Phan VB, Nguyen TT, Bryla D, Tran CT, et al. The epidemiology of typhoid fever in the Dong Thap Province, Mekong Delta region of Vietnam. The American journal of tropical medicine and hygiene. 2000 May;62(5):644–8. doi:10.4269/ajtmh.2000.62.644
12. Sinha A, Sazawal S, Kumar R, Sood S, Reddaiah VP, Singh B, et al. Typhoid fever in children aged less than 5 years. The Lancet. 1999 Aug;354(9180):734–7. doi:10.1016/s0140-6736(98)09001-1
13. Brooks WA, Hossain A, Goswami D, Sharmeen AT, Nahar K, Alam K, et al. Bacteremic typhoid fever in children in an urban slum, Bangladesh. Emerging Infectious Diseases. 2005 Feb;11(2):326–9. doi:10.3201/eid1102.040422
14. Simanjuntak CH, Totosudirjo H, Haryanto P, Suprijanto E, Paleologo FP, Punjabi NH, et al. Oral immunisation against typhoid fever in Indonesia with TY21A vaccine. The Lancet. 1991 Oct;338(8774):1055–9. doi:10.1016/0140-6736(91)91910-m
