# Supplementary material for: Evaluating the impact and cost-effectiveness of typhoid conjugate vaccine schedules across diverse settings: A multi-model comparison
Source: PLoS Med. 2026 Oct 13;23(10):e1005280. doi: 10.1371/journal.pmed.1005280 (PMC13686463; doi:10.1371/journal.pmed.1005280)
Supplement: Supplementary file 6 [file pmed.1005280.s005.docx]

***S3 Supplementary Text: Vaccine Effectiveness and Waning***

**Evaluating the Impact and Cost-Effectiveness of Typhoid Conjugate Vaccine Schedules Across Diverse Settings: A Multi-Model Comparison**

*Authors:*

Catherine G.C. Wenger^1,&^, Kyra H. Grantz^2,&^, Tigist F. Menkir^3,&^, Alina M. Muellenmeister^4,&^, Zeaan Pithawala^1^, Raymond Hutubessy^5^, Vittal Mogasale^5^, Alicia N.M. Kraay^2^, Nick Scott^4^, Romesh G. Abeysuriya^4,$^, Jason R. Andrews^3,$^, Jillian Gauld^2,$^, Nathan C. Lo^3,$^, Virginia E. Pitzer^1,$^

*Affiliations:*

1 Department of Epidemiology of Microbial Diseases, Yale School of Public Health, Yale University, New Haven, Connecticut, United States of America

2 Institute for Disease Modeling, Gates Foundation, Seattle, Washington, United States of America

3 Division of Infectious Diseases and Geographic Medicine, Department of Medicine, Stanford University, Stanford, California, United States of America

4 Burnet Institute, Melbourne, Victoria, Australia

5 Department of Performance, Finance and Delivery, World Health Organization, Geneva, Switzerland

^&^co-first authors

^$^co-last authors

Disclaimer: RH and VM are staff members of the World Health Organization. The views expressed are their personal ones and do not necessarily represent the views of their organization. KHG, AK, and JG are employees of the Gates Foundation; however, this study does not necessarily represent the views of the Gates Foundation.

# A. Vaccine Effectiveness and Waning

We considered two different scenarios for the waning of vaccine effectiveness (VE) over time based on observations from randomized controlled trials (RCTs). The differences in the waning of VE between RCTs conducted in Malawi and Bangladesh may be due to differences in the local disease dynamics—potentially transmission intensity—although the true cause is unknown. Therefore, we model both fast- and slow-waning scenarios to understand how the preferred vaccination strategy may change based on waning assumptions.

The “slow-waning” scenario is based on data from the TyVAC-Malawi RCT conducted in Blantyre, Malawi [1,2]. Trial participants 9 months to 12 years of age were randomized to receive either the Tybar-TCV typhoid conjugate vaccine (Bharat Biotech) or a control vaccine (meningococcal A conjugate vaccine) and were followed for up to 4.61 years after vaccination [1]. The overall VE for all age groups in the intention-to-treat analysis declined from 80.7% (95% confidence interval (CI) [62.4%, 89.6%]) after 2 years of follow-up [1] to 78.3% (95% CI [66.3%, 86.1%]) after 4-5 years of follow-up [2], suggesting minimal waning of immunity among study participants. Data was available to stratify participants into two age groups based on age at vaccination: 9 months to <5 years and 5 to <15 years of age [1,2].

The “fast-waning” scenario is based on data from the TyVAC-Bangladesh cluster RCT and TyVOID study conducted in Dhaka, Bangladesh [3,4]. Beginning in April 2018, the TyVAC-Bangladesh trial randomized participants 9 months to <16 years of age living in 150 geographic clusters to either receive the Tybar-TCV (Bharat Biotech) or a control vaccine (SA 14-14-2 against Japanese encephalitis) based on their cluster of residence. Participants were followed for 17.1 months on average until December 2019. Total vaccine effectiveness for all age in the TyVAC-Bangladesh trial was 85% (97.5% CI [76%, 91%]) [3]. Data was available to stratify participants into three age groups based on age at vaccination: 9 months to <2 years, 2 years to <5 years, and 5 to 15 years of age [3,4].

Following the trial (and a hiatus in surveillance due to the COVID-19 pandemic), participants who had received the control vaccine were offered the Tybar-TCV vaccine. The TyVOID study then followed the recent TCV recipients (vaccinated in 2021) and compared them to previous TCV recipients (vaccinated in 2018-19) to estimate relative vaccine efficacy as well as conducted a test-negative case-control study comparing the vaccination status of those who test blood-culture-positive for *Salmonella* Typhi to those who tested negative or positive for another organism [4]. The VE for all age groups in the previous-TCV group 3 to 5 years after vaccination was 55% (95% CI [36%, 68%]) based on the test-negative design, which was similar to the VE of 50% (95% CI [-13%, 78%]) extrapolated from the relative efficacy among the previous versus recent TCV recipients [4]. Follow-up has continued, and VE has declined further based on data through May 2025. The VE estimates by age group and duration of follow-up are available in Table A.1.

**Table A.1: Vaccine efficacy and effectiveness estimates by age group and duration of follow-up for the TyVAC-Malawi, TyVAC-Bangladesh and TyVOID-Bangladhesh studies.** Values are presented as the mean estimates and 95% confidence intervals, except for the first follow-up period in Bangladesh, for which 97.5% confidence intervals were reported in the study [3].

| ***Malawi*** | | Follow-up period | | | |
| --- | --- | --- | --- | --- | --- |
| Midpoint of follow-up | | 1 year | | 2.305 years | |
| Age group | <5 years | 74.4% (31.7%, 90.4%) | | 70.6% (6.4%, 93%) | |
|  | 5-15 years | 83.7% (63.6%, 92.7%) | | 79.3% (63.5%, 89.0%) | |
| ***Bangladesh*** | | Follow-up period | | | |
| Midpoint of follow-up | | 1 year | 4 years | | 6 years |
| Age group | <2 years | 81% (39%, 94%) | 24% (-29%, 55%) | | -5% (-118%, 49%) |
|  | 2-<5 years | 80% (62%, 89%) | 59% (12%, 81%) | | 14% (-196%, 75%) |
|  | 5-15 years | 88% (78%, 93%) | 74% (41%, 89%) | | 32% (-63%, 71%) |

To estimate age-specific initial vaccine efficacy and mean duration of protection from the VE estimates in Table 1, we used Bayesian inference implemented via Markov Chain Monte Carlo using the JAGS (Just Another Gibbs Sampler) software. The parameters were jointly estimated using an observational model and a time-varying protection model using uninformative prior distributions.

For both waning scenarios, we evaluated different models assuming vaccine protection follows an exponential distribution, a gamma distribution with uninformative shape and scale parameters, and gamma distribution with fixed shape parameters at $s$ = 2, 3, 5, 7, and 10; note that the exponential distribution is equivalent to a gamma distribution with a shape parameter of $s=1$. We compared the models based on Akaike information criteria (AIC), Bayesian information criteria (BIC), and deviance information criteria (DIC) and chose the most parsimonious fit.

For the slow-waning scenario, the expected VE for those vaccinated in age group *a* at time *t* post-vaccination is described by the exponential waning function:

$${VE}_{a}\left( t \right)={VE}_{0,a}exp({-\omega}_{v,a}t)$$

where 𝑉𝐸_0,𝑎_ is the initial vaccine efficacy, which represents the reduction in risk of infection for a TCV recipient shortly after vaccination (before any waning has occurred), and the duration of TCV protection is exponentially distributed with mean 1/𝜔_𝑣,𝑎_, where 𝜔_𝑣,𝑎_ is the rate of waning of TCV-induced immunity in age group *a*.

For the fast-waning scenario, the expected VE for those vaccinated in age group *a* at time *t* post-vaccination is described by the function:

$${VE}_{a}\left( t \right)={VE}_{0,a}\times\left[ 1-F_{\Gamma}(t,s,\omega_{v,a}) \right]$$

where 𝑉𝐸_0,𝑎_ is again the initial vaccine efficacy, and the duration of TCV protection follows a gamma distribution with a fixed shape parameter $s$, where 𝜔_𝑣,𝑎_ is the rate of waning of TCV-induced immunity in age group *a*. The overall best performing model was identified to be the model with shape $s=2$.

We used an observational model to account for uncertainty in the VE estimates, given that the trials were conducted in a limited sample of the full population. We defined the likelihood of the observed values of 𝑉𝐸_𝑎_(𝑡) from Table 1, given the “true” level of protection and the precision of the trial measurements. Since relative risk (=1-VE) follows an approximately normal distribution once it is log-transformed, we modeled:

$$\log\left( 1-\hat{VE}\left( t \right) \right)\sim Normal(log(1-VE\left( t \right),\sigma^{2})$$

where the variance, 𝜎^2^, can be approximated from the confidence interval of the observed VE, $\hat{VE}\left( t \right)$.

The fitted models are shown in Figure A.1. Two models (IDM and Yale) used these estimates directly in the simulations, while two models (Burnet and Stanford) directly fit their models to the clinical trial data. See S5 Text Sections B and D for a description of the fitting approaches.

**Table A.2: Estimated vaccine effectiveness and waning parameters for the transmission models.** Values are presented as the mean estimates and 95% credible intervals.

| Average duration of protection (years) | | | |
| --- | --- | --- | --- |
| Group | **Burnet** | **Stanford** | **IDM/Yale** |
| *Fast waning* | | | |
| <2y | 1.82 | 1.1 (1.07-1.17) | 2.8 (0.01-6.6) |
| 2 to <5y | 4.2 | 7.0 (2.7-18.5) | 7.3 (2.6-34.0) |
| >5y | 5.1 | 7.1 (5.2-10.6) | 10.7(4.2-42.3) |
| *Slow waning* | | | |
| <5y | 7.2 | 73.9 (41.2-83.3) | 46.4 (3.7-97.3) |
| >5y | 7.2 | 36.9 (21.8-81.4) | 52.4 (10.4-97.6) |
| VE0 | | | |
| Group | **Burnet** | **Stanford** | **IDM/Yale** |
| *Fast waning* | | | |
| <2y | 85% | 99%  (99%,100%) | 85.8%  (85.3%, 86.3%) |
| 2 to <5y | 78% | 82.8%  (71.2%, 97.2%) | 82.7%  (82.3%, 83.1%) |
| >5y | 80% | 95.6%  (92.8%, 97.6%) | 89.3%  (89.0%, 89.6%) |
| *Slow waning* | | | |
| <5y | 75% | 89.0%  (86.8%, 90.7%) | 80.2%  (79.7%, 80.6%) |
| >5y | 75% | 93.6%  (89.4%, 95.8%) | 84.1%  (83.8%, 84.3%) |

y = years

**Figure A.1: Modeled statistical estimates of waning of vaccine effectiveness over time by age group**. The lines represent the modeled vaccine protection, while the shaded regions correspond to the 95% credible intervals for the slow-waning scenario (blue) and fast-waning scenario (red). The points with vertical lines are the observed vaccine effectiveness (VE) estimates and 95% confidence intervals from the randomized controlled trials.

b.

c.

a.

**References**

1. Patel PD, Patel P, Liang Y, Meiring JE, Misiri T, Mwakiseghile F, et al. Safety and efficacy of a typhoid conjugate vaccine in Malawian children. New England Journal of Medicine. 2021 Sept 16;385(12):1104–15. doi:10.1056/nejmoa2035916
2. Patel PD, Liang Y, Meiring JE, Chasweka N, Patel P, Misiri T, et al. Efficacy of typhoid conjugate vaccine: Final analysis of a 4-year, phase 3, randomised controlled trial in malawian children. The Lancet. 2024 Feb;403(10425):459–68. doi:10.1016/s0140-6736(23)02031-7
3. Qadri F, Khanam F, Liu X, Theiss-Nyland K, Biswas PK, Bhuiyan AI, et al. Protection by vaccination of children against typhoid fever with a VI-tetanus toxoid conjugate vaccine in urban Bangladesh: A cluster-randomised trial. The Lancet. 2021 Aug;398(10301):675–84. doi:10.1016/s0140-6736(21)01124-7
4. Qadri F, Khanam F, Zhang Y, Biswas PK, Voysey M, Mujadidi YF, et al. 5-year vaccine protection following a single dose of vi-tetanus toxoid conjugate vaccine in Bangladeshi children (Tyvoid): A cluster randomised trial. The Lancet. 2024 Oct;404(10461):1419–29. doi:10.1016/s0140-6736(24)01494-6
