# Supplementary material for: Evaluating the impact and cost-effectiveness of typhoid conjugate vaccine schedules across diverse settings: A multi-model comparison
Source: PLoS Med. 2026 Oct 13;23(10):e1005280. doi: 10.1371/journal.pmed.1005280 (PMC13686463; doi:10.1371/journal.pmed.1005280)
Supplement: Supplementary file 7 [file pmed.1005280.s006.docx]

***S4 Supplementary Text: Vaccine Coverage***

**Evaluating the Impact and Cost-Effectiveness of Typhoid Conjugate Vaccine Schedules Across Diverse Settings: A Multi-Model Comparison**

*Authors:*

Catherine G.C. Wenger^1,&^, Kyra H. Grantz^2,&^, Tigist F. Menkir^3,&^, Alina M. Muellenmeister^4,&^, Zeaan Pithawala^1^, Raymond Hutubessy^5^, Vittal Mogasale^5^, Alicia N.M. Kraay^2^, Nick Scott^4^, Romesh G. Abeysuriya^4,$^, Jason R. Andrews^3,$^, Jillian Gauld^2,$^, Nathan C. Lo^3,$^, Virginia E. Pitzer^1,$^

*Affiliations:*

1 Department of Epidemiology of Microbial Diseases, Yale School of Public Health, Yale University, New Haven, Connecticut, United States of America

2 Institute for Disease Modeling, Gates Foundation, Seattle, Washington, United States of America

3 Division of Infectious Diseases and Geographic Medicine, Department of Medicine, Stanford University, Stanford, California, United States of America

4 Burnet Institute, Melbourne, Victoria, Australia

5 Department of Performance, Finance and Delivery, World Health Organization, Geneva, Switzerland

^&^co-first authors

^$^co-last authors

Disclaimer: RH and VM are staff members of the World Health Organization. The views expressed are their personal ones and do not necessarily represent the views of their organization. KHG, AK, and JG are employees of the Gates Foundation; however, this study does not necessarily represent the views of the Gates Foundation.

# A. Vaccine Coverage

The World Health Organization (WHO) has limited data on typhoid conjugate vaccine (TCV) coverage for countries, particularly within the African (AFR) and Western Pacific (WPR) regions [1]. Therefore, we used coverage estimates for more widely used vaccines that are administered at time points of interest for potential typhoid vaccination schedules. For routine vaccination at 9 months and 15 months, we used WHO/UNICEF Estimates of National Immunization Coverage (WUENIC) estimates for measles-containing vaccine first and second dose (MCV1 and MCV2), respectively, for low- and lower-middle-income countries [2]. The MCV1 estimates were similar to coverage estimates for TCVs in countries that introduced routine immunization [1,2]. To estimate coverage for routine vaccination at 2 years of age (which is outside the regular Expanded Programme on Immunization [EPI] schedule), MCV2 estimates were multiplied by a fraction (randomly sampled uniformly from 0.5 to 1), which assumes it is harder to reach a child for vaccination at 2 years. For routine vaccination at 5 years of age and booster doses at 5 and 10 years, we assumed coverage to be some fraction (again randomly sampled uniformly from 0.5 to 1) of UNICEF school attendance estimates [3], since we assume that vaccine delivery at this age would likely occur through a school-based program. Finally, catch-up campaign coverage was based on estimates of MCV supplementary immunization activity (SIA) coverage. Catch-up campaigns are typically immunization events where the coverage achieved depends on a one-time mobilization of the health system to actively reach children; coverage estimates for TCV catch-up campaigns administered to date have been similar to that of MCV campaigns.

Coverage estimates were matched by country, sampled with replacement, and used for each simulation to preserve correlation between coverage types while allowing for parameter uncertainty. We only included countries that fell within the WHO regions of interest (Africa, Asia, Western Pacific) and had coverage estimates for all vaccine schedules.

**Table A.1: Mean and 95% confidence intervals (CIs) of vaccine coverage estimates.** MCV1 and MCV2 are the first and second doses of the measles-containing vaccine schedule. Values are reported as mean estimates and 95% confidence intervals (CIs).

| Vaccine Schedule | Mean Coverage (95% CI) |
| --- | --- |
| Supplementary Immunization Activities | 86.8% (82.8%, 90.9%) |
| MCV1 | 78.4% (74.0%, 82.8%) |
| MCV2 | 64.3% (57.9%, 70.6%) |
| School attendance | 80.5% (76.4%, 84.6%) |

**Figure A.1: Distribution of vaccine coverage estimates.** MCV1 and MCV2 are the first and second doses of the measles-containing vaccine schedule.

# References

1. WHO. WHO immunization data portal - Typhoid vaccination coverage [Internet]. World Health Organization; 2021 [cited 2026 Feb 11]. Available from: <https://immunizationdata.who.int/global/wiise-detail-page/typhoid-vaccination-coverage?ANTIGEN=TYPHOID_CONJ&YEAR=>
2. World Health Organization. Measles vaccination coverage [Internet]. World Health Organization; [cited 2025 Dec 11]. Available from: https://immunizationdata.who.int/global/wiise-detail-page/measles-vaccination-coverage
3. UNICEF. Education overview: Adjusted net attendance rates [Internet]. 2022 [cited 2025 Dec 12]. Available from: <https://data.unicef.org/topic/education/overview/>
4. Gharpure R, Longley AT, Takamiya M, Hidle A, Munyanyi M, Chawurura T, Maxwell L, Mamire G, Chaora G, Chakauya J, Rupfutse M, Poncin M, Gasasira A, Date K, Manangazira P, Sreenivasan N. Typhoid conjugate vaccine perceptions and coverage among children and adults: Findings from a post-campaign coverage survey - Harare, Zimbabwe, 2019. Vaccine. 2024 Oct 24;42(24):126086.
