# Supplementary material for: Evaluating the impact and cost-effectiveness of typhoid conjugate vaccine schedules across diverse settings: A multi-model comparison
Source: PLoS Med. 2026 Oct 13;23(10):e1005280. doi: 10.1371/journal.pmed.1005280 (PMC13686463; doi:10.1371/journal.pmed.1005280)
Supplement: Supplementary file 8 [file pmed.1005280.s007.docx]

***S5 Supplementary Text: Model Descriptions***

**Evaluating the Impact and Cost-Effectiveness of Typhoid Conjugate Vaccine Schedules Across Diverse Settings: A Multi-Model Comparison**

*Authors:*

Catherine G.C. Wenger^1,&^, Kyra H. Grantz^2,&^, Tigist F. Menkir^3,&^, Alina M. Muellenmeister^4,&^, Zeaan Pithawala^1^, Raymond Hutubessy^5^, Vittal Mogasale^5^, Alicia N.M. Kraay^2^, Nick Scott^4^, Romesh G. Abeysuriya^4,$^, Jason R. Andrews^3,$^, Jillian Gauld^2,$^, Nathan C. Lo^3,$^, Virginia E. Pitzer^1,$^

*Affiliations:*

1 Department of Epidemiology of Microbial Diseases, Yale School of Public Health, Yale University, New Haven, Connecticut, United States of America

2 Institute for Disease Modeling, Gates Foundation, Seattle, Washington, United States of America

3 Division of Infectious Diseases and Geographic Medicine, Department of Medicine, Stanford University, Stanford, California, United States of America

4 Burnet Institute, Melbourne, Victoria, Australia

5 Department of Performance, Finance and Delivery, World Health Organization, Geneva, Switzerland

^&^co-first authors

^$^co-last authors

Disclaimer: RH and VM are staff members of the World Health Organization. The views expressed are their personal ones and do not necessarily represent the views of their organization. KHG, AK, and JG are employees of the Gates Foundation; however, this study does not necessarily represent the views of the Gates Foundation.

Table of Contents

[Model Descriptions	3](#_Toc241485405)

[A. Model harmonization	3](#_Toc241485406)

[B. Burnet Institute	4](#_Toc241485407)

[C. Institute for Disease Modeling (IDM)	9](#_Toc241485408)

[D. Stanford University	12](#_Toc241485409)

[E. Yale University	15](#_Toc241485410)

[References	18](#_Toc241485411)

# Model Descriptions

## A. Model harmonization

For this multi-model comparison, four modeling groups were selected in response to a “Request for Proposals for Modelled Evidence To Support A Strategic Advisory Group of Experts Working Group (SAGE WG) On Typhoid Conjugate Vaccine (TCV)” [1]; the teams were from the Burnet Institute, the Bill & Melinda Gates Foundation Institute for Disease Modeling (IDM), Stanford University, and Yale University. All four models are age-stratified and simulate typhoid transmission dynamics in a population; two are compartmental (Stanford and Yale) and two are agent-based (Burnet and IDM). We harmonized the models around key features of typhoid natural history while preserving unique model elements where there may be uncertainty about typhoid dynamics. In all models, susceptible individuals become infected based on the force of infection of typhoid in the population at that time step, which is proportional to the prevalence of infectious individuals. Following a period of symptomatic or asymptomatic infection, individuals may recover and have natural immunity or become chronic carriers, asymptotically contributing to the force of infection. Natural immunity is modeled as either a recovered class whose protection wanes until they enter the fully susceptible class or a partially susceptible class with a certain level of protection based on previous infections. We model clinical and subclinical infection based on prior infection history, where individuals may or may not experience symptoms, but both groups contribute to the force of infection.

All models assume individuals can also gain immunity via vaccination. The Yale, IDM, and Burnet models assume fully “leaky” vaccine-induced immunity against infection in which individuals enter the vaccinated class based on vaccine coverage. Upon exposure to typhoid, a fraction of those individuals, equal to the initial vaccine efficacy (*VE*_0,_*_a_*) in age group *a* times the number of vaccinated individuals in age group *a*, remain protected, while the complement can become infected and move to the infected state. The Stanford model assumed a hybrid leaky/all-or-nothing vaccine-induced immunity mechanism, with separate protection against infection versus disease, as described in section 4. For all models, vaccine-derived immunity is assumed to wane at a constant rate over time (*ω_v,a_*); when protection has fully waned, individuals re-enter a susceptible state.

We first calibrated the models to the harmonized demographic data by fitting the age-specific background mortality, establishing similar, stable population dynamics in each model. Then, each model was fitted to the age-specific incidence data from the three incidence settings. This was achieved through estimating the model-specific transmission parameters (see below). The models also simulate vaccine efficacy and waning of vaccine protection as described in the S3 Text. We assumed booster doses are administered only to individuals who previously received a dose during routine immunization or a catch-up campaign.

## B. Burnet Institute

**Figure B.1: Model structure for the Burnet agent-based model**

FOI = force of infection

The Burnet group developed an agent-based model using the *Starsim* framework to simulate typhoid disease dynamics. Diffuse direct transmission is modelled as a proxy for all sources of transmission, including environmental transmission. In this way, the overall force of infection is captured without explicitly modelling each individual transmission route or requiring detailed data on specific pathways. Beyond the common features among all the models, infected individuals in this model may become chronic carriers based on age- and sex-specific probabilities and are assumed to have life-long carriage. Natural and vaccine immunity provide leaky protection against both infection and symptoms, and any prior infection is assumed to be fully protective against subsequent acute infections.

To reproduce the incidence of each incidence setting, we estimated the transmission coefficient, duration of natural immunity, relative infectiousness of chronic carriers, age-specific relative risk of infection, and the symptomatic fraction of primary infections. We calibrated the model by adjusting parameters such as the transmission rate or probability of an acute infection to minimize the differences between the model projections and archetypal acute incidence data. For each burden setting, the model was calibrated to a steady state that matched the observed age-specific incidence of typhoid. To introduce a level of uncertainty (10%) into our calibrated set of parameters, we drew samples from distributions centered around the parameter values, with standard deviations set to 10% of their respective means. Because model simulations can yield a broad range of outcomes for a given set of key parameters, we filtered simulations based on how realistically they represented the observed data. Specifically, for each age group, the mean incidence over the analysis period was required to fall within 25% of the observed age-specific incidence, and the trend in incidence (i.e., the slope) over this same period could not exceed 25%. Figure B.2 compares age-specific typhoid incidence per 100,000 person-years across the baseline incidence settings (medium, high, and very high) with the modelled incidence, illustrating the fit of the model to the observed data for each age group.

Additionally, the model was fitted under slow and fast vaccine immunity waning scenarios using data from the TyVAC Malawi [2,3] and TyVOID Bangladesh [4,5] studies, respectively. We estimated waning rates by explicitly simulating the trial cohort structure and vaccine coverage, fitting VE_0_ and waning rates such that the model’s incidence-based estimates of vaccine efficacy matched the observed trial data (Figures B.3 and B.4). In the fast-waning scenario, this adjustment involved age-group-specific efficacy and waning rates. The infectiousness of chronic carriers was calibrated to align with the observed indirect protection in the TyVAC-Bangladesh cluster randomized controlled trial (cRCT).

**Table B.1 Fitted and calibrated parameters by incidence setting for the Burnet agent-based model.** Values are presented as the median and inter-quartile range.

| **Medium** | | |
| --- | --- | --- |
| Transmission Rate | 0.2542 (0.2426 – 0.2664) |  |
| Probability of acute infection | 0.2966 (0.2770 – 0.3168) |  |
| Duration of natural immunity | 4.9533 (4.6443 – 5.3030) |  |
| Relative infectiousness of chronic carriers | 0.1970 (0.1854 – 0.2099) |  |
| Relative susceptibility of 0 to 2-year-olds | 0.3359 (0.3140 – 0.3591) |  |
| Relative susceptibility of 2 to 5-year-olds | 0.7014 (0.6627 – 0.7440) |  |
| Relative susceptibility of 5 to 10-year-olds | 0.9230 (0.8658 – 0.9792) |  |
| Relative susceptibility of 10 to 15-year-olds | 1 (1 – 1) |  |
| Relative susceptibility of >15-year-olds | 0.5319 (0.5022 – 0.5641) |  |
| **High** | | |
| Transmission Rate | 0.3357 (0.3210 – 0.3493) |  |
| Probability of acute infection | 0.3509 (0.3292 – 0.3733) |  |
| Duration of natural immunity | 5.1090 (4.7538 – 5.4584) |  |
| Relative infectiousness of chronic carriers | 0.1977 (0.1860 – 0.2089) |  |
| Relative susceptibility of 0 to 2-year-olds | 0.4111 (0.3879 – 0.4341) |  |
| Relative susceptibility of 2 to 5-year-olds | 1.2661 (1.1902 – 1.3391) |  |
| Relative susceptibility of 5 to 10-year-olds | 1.0520 (0.9888 – 1.1055) |  |
| Relative susceptibility of 10 to 15-year-olds | 1 (1 – 1) |  |
| Relative susceptibility of >15-year-olds | 0.4180 (0.3932 – 0.4420) |  |
| **Very High** | | |
| Transmission Rate | 0.4665 (0.4515 – 0.4814) |  |
| Probability of acute infection | 0.6380 (0.6011 – 0.6712) |  |
| Duration of natural immunity | 5.0950 (4.7562 – 5.4612) |  |
| Relative infectiousness of chronic carriers | 0.1990 (0.1868 – 0.2133) |  |
| Relative susceptibility of 0 to 2-year-olds | 0.3026 (0.2839 – 0.3211) |  |
| Relative susceptibility of 2 to 5-year-olds | 1.1031 (1.0309 – 1.1739) |  |
| Relative susceptibility of 5 to 10-year-olds | 0.9930 (0.9307 – 1.0598) |  |
| Relative susceptibility of 10 to 15-year-olds | 1 (1 – 1) |  |
| Relative susceptibility of >15-year-olds | 0.9209 (0.8632 – 0.9842) |  |

**Figure B.2: Model fit to the incidence data for the Burnet agent-based model** (a) Model-predicted absolute acute incidence (bars including uncertainty ranges) by age group over 20 years, with chronic carrier prevalence overlaid (red line) and (b) Comparison of model-predicted incidence (black crosses including uncertainty ranges) with observed data (bars) for medium (left), high (middle), and very high (right) incidence settings.

**Figure B.3: Model fit to the Bangladesh vaccine efficacy data for the Burnet agent-based model** Total, overall, and indirect vaccine protection over time after campaign introduction by age group. Points show model predictions (median and 5^th^ – 95^th^ percentile ranges) and observed data, with the dashed line representing the input vaccine effectiveness (VE).

**Figure 2.4: Model fit to the Malawi vaccine efficacy data for the Burnet agent-based model** Vaccine efficacy over time after campaign introduction by age group. Lines show model predictions (median and 5^th^ – 95^th^ percentile ranges) and observed data, with the grey dashed line representing the input vaccine effectiveness (VE).

## C. Institute for Disease Modeling (IDM)

**Figure C.1: Model structure for the IDM agent-based model** Black arrows show the movement of individuals, and blue dashed lines show shedding into the environment. CFU is colony-forming unit.

The IDM model, Typhoidsim, is an agent-based model developed in the *Starsim* platform as an update to the previously developed EMOD (Epidemiological MODeling software) model [6]. Individuals in this model are immune to infection upon birth due to maternal immunity and/or reduced environmental exposure. Individuals enter the susceptible population with an age-specific probability, governed by an exponential curve whose concavity is a fitted model parameter and a fitted maximum age by which all individuals are considered susceptible.

Individuals can be infected via long-cycle transmission through exposure to environmental reservoirs, which is mediated by a dose-response curve. The prevalence of bacteria in the environment is also subject to seasonal attenuation, which was fit to data from Chile in a previous iteration of the model [6]. Subsequent infections occur at a reduced rate based on the number of prior infections an individual has experienced. After infection, individuals experience a latent period that lasts an average of 2 days before progressing to either subclinical or clinical infection. After infection, individuals may become lifelong chronic carriers at a rate depending on age- and sex-specific risk of gallstone disease. Latent, incubating infections (relative risk, 0.5) and chronic carriers (relative risk, 0.24) are assumed to be less infectious relative to subclinical or clinical infections. Parameters governing acute infectiousness of clinical infections, rate of susceptible introduction, natural immunity, and symptomatic infection were fit to the age distribution and overall magnitude of incidence in each archetypal setting (Table C.1).

Individuals in any model state are eligible for vaccination. Vaccination is modeled as reducing susceptibility to infection by an amount proportional to age-specific vaccine efficacy (“leaky vaccination”). Vaccine protection was modeled as a gamma decay function (shape = 1 for slow waning, shape = 2 for fast waning), with individually-varying, age-specific initial response and duration of protection simulated from posterior samples generated by the statistical model of VE and waning parameters described in the S3 Text. Vaccine coverage varied across stochastic iterations of the model, sampled from country-specific proxy vaccination data described in the S4 Text.

To validate transmission and vaccination dynamics, we simulated routine immunization at 9 months and a one-time vaccination campaign of individuals 9 months to 15 years of age to reflect the conditions of the Bangladesh cRCT. We also simulated an observation process to match the age-specific person-time distribution of the cRCT. We estimated overall, total, and indirect vaccine protection in this simulated cohort and confirmed that these values were within the cRCT’s confidence intervals.

**Table C.1 Fitted and calibrated parameters by incidence setting for the IDM agent-based model**

| **Medium** | | |
| --- | --- | --- |
| Acute infectiousness | 3788.9 |  |
| Susceptible introduction: concavity | 3.068 |  |
| Susceptible introduction: maximum age | 15 |  |
| Protection via natural immunity per prior infection | 0.871 |  |
| Probability of symptomatic infection | 0.025 |  |
| **High** | | |
| Acute infectiousness | 4925.1 |  |
| Susceptible introduction: concavity | -0.200 |  |
| Susceptible introduction: maximum age | 3 |  |
| Protection via natural immunity per prior infection | 0.815 |  |
| Probability of symptomatic infection | 0.098 |  |
| **Very High** | | |
| Acute infectiousness | 9,713.6 |  |
| Susceptible introduction: concavity | -0.259 |  |
| Susceptible introduction: maximum age | 3 |  |
| Protection via natural immunity per prior infection | 0.812 |  |
| Probability of symptomatic infection | 0.398 |  |

**Figure C.2: Model fit to the incidence data for the IDM agent-based model.**

## D. Stanford University

**Figure D.1: Model structure for the Stanford compartmental model**

Stanford developed a compartmental model that simulates direct typhoid transmission at the population level, where individuals become infected at a rate related to the number of infectious persons and enter symptomatic (Is) and asymptomatic (Ia) compartments based on an age-specific symptomatic fraction. Based on the robustness of the immune response, individuals may recover with natural immunity (R) for some time (after which they become re-susceptible to infection), return directly to the susceptible class (S), or become a chronic carrier (C) with a duration of 10 years. Vaccine protection is modeled through an initial all-or-nothing component where vaccinated individuals either obtain protection (V) against symptomatic disease or have no protection (Vw). Individuals who have vaccine protection may become asymptomatically infected (Iv,a), simulating a leaky component of vaccine protection. Protected individuals wane into the Vw compartment upon losing vaccine immunity, where they are susceptible to both symptomatic and asymptomatic infection, but can have their protection restored to initial levels by a booster dose. To ensure demographic stability in the absence of disease transmission, we used Nelder-Mead optimization to calibrate age-specific population sizes, background mortality rates, and the overall birth rate. We also applied Nelder-Mead optimization to calibrate our natural history parameters, i.e., the age-specific transmission rates, symptomatic fractions, and an overall duration of natural immunity.

The vaccine-associated compartments are governed by several key parameters, namely the risk of asymptomatic infection among those in V, the reduction in infectiousness from asymptomatically infected previously vaccinated individuals compared to the vaccine-naïve, and age-specific vaccine effectiveness (VE) and waning rates. To appropriately capture waning within our model framework and recreate indirect protection, we calibrated our model using equilibrium incidence data from the very high incidence archetype, to two sets of targets: 1) 19% indirect protection and 2) vaccine effectiveness in the different age groups at their trial-reported time intervals. We did this for the fast- and slow-waning scenarios separately. VE and indirect targets were calibrated concurrently using bounded Black-Box optimization, applying the *BlackBoxOptim* package in Julia. For the fast-waning scenario, we calibrated to VE targets for the youngest and oldest of the three age categories for which VE data were available, and fixed VE to its original value for the middle age group, which consisted of only one age. For the slow-waning scenario, we calibrated to VE targets for the two age categories for which VE data were available.

**Table D.1 Fitted and calibrated parameters by incidence setting for the Stanford compartmental model**

| **Medium** | | |
| --- | --- | --- |
| Duration of natural immunity | 18.0 years |  |
| Transmission rate or beta (age group 1) | 3.60 /month |  |
| Transmission rate or beta (age group 2) | 3.68 /month |  |
| Transmission rate or beta (age group 3) | 5.09/month |  |
| Transmission rate or beta (age group 4) | 5.80/month |  |
| Transmission rate or beta (age group 5) | 0.845/month |  |
| Symptomatic fraction (age group 1) | 0.00573 |  |
| Symptomatic fraction (age group 2) | 0.0128 |  |
| Symptomatic fraction (age group 3) | 0.0135 |  |
| Symptomatic fraction (age group 4) | 0.0139 |  |
| Symptomatic fraction (age group 5) | 0.0404 |  |
| **High** | | |
| Duration of natural immunity | 19.4 years |  |
| Transmission rate or beta (age group 1) | 4.70/month |  |
| Transmission rate or beta (age group 2) | 8.52/month |  |
| Transmission rate or beta (age group 3) | 6.47/month |  |
| Transmission rate or beta (age group 4) | 3.85/month |  |
| Transmission rate or beta (age group 5) | 0.691/month |  |
| Symptomatic fraction (age group 1) | 0.0175 |  |
| Symptomatic fraction (age group 2) | 0.0346 |  |
| Symptomatic fraction (age group 3) | 0.0442 |  |
| Symptomatic fraction (age group 4) | 0.0612 |  |
| Symptomatic fraction (age group 5) | 0.117 |  |
| **Very High** | | |
| Duration of natural immunity | 6.5 years |  |
| Transmission rate or beta (age group 1) | 2.52/month |  |
| Transmission rate or beta (age group 2) | 18.5/month |  |
| Transmission rate or beta (age group 3) | 12.1/month |  |
| Transmission rate or beta (age group 4) | 7.52/month |  |
| Transmission rate or beta (age group 5) | 0.993/month |  |
| Symptomatic fraction (age group 1) | 0.0475 |  |
| Symptomatic fraction (age group 2) | 0.0529 |  |
| Symptomatic fraction (age group 3) | 0.0754 |  |
| Symptomatic fraction (age group 4) | 0.0729 |  |
| Symptomatic fraction (age group 5) | 0.0664 |  |

**Figure D.2: Model fit to the incidence data for the Stanford compartmental model**

## E. Yale University

**Figure E.1: Model structure for the Yale compartmental model**

The Yale model is a compartmental transmission dynamic model that models primary and secondary infections, which are mediated by the force of infection and age-specific risk of infection. Primary infections may be clinical or asymptomatic based on the symptomatic fraction. All secondary and subsequent infections are assumed to be asymptomatic but contribute to the force of infection. Infected individuals may recover and be protected by natural immunity for an assumed duration of 2 years or become a life-long chronic carrier that fractionally contributes to the force of infection. Any individual may be vaccinated, but only those in the susceptible and recovered classes receive protection from the vaccine. All vaccinated and protected individuals enter a vaccine class according to their age group, where they are protected until they become infected due to a leaky vaccine assumption or become susceptible after their immunity wanes. Those vaccinated while in the infected or carrier compartments (I1, I2, C) move to the corresponding vaccinated compartments (Iv1, Iv2, C). We fit the demographic and incidence data via Maximum Likelihood Estimation using a multinomial distribution for population age structure and a binomial distribution for age-specific incidence. Table E.1 details the fitted incidence parameters, and Figure E.2 shows the model fit to the age-specific incidence data by incidence setting.

**Table E.1 Fitted and calibrated parameters by incidence setting for the Yale compartmental model.**

| **Medium** | | |
| --- | --- | --- |
| R0 Equation (dependent on rC) | 2.986 + 18.299rC + -10.847rC^2 |  |
| Relative risk of infection for 0 to 2-year-olds | 0.23 |  |
| Relative risk of infection for 2 to 5-year-olds | 0.57 |  |
| Probability of symptomatic primary infection | 0.03 |  |
| Relative infectiousness of chronic carriers (rC) | Beta(8.34,36.08) |  |
| **High** | | |
| R0 Equation (dependent on rC) | 2.341 + 34.351rC + -18.846rC^2 |  |
| Relative risk of infection for 0 to 2-year-olds | 0.25 |  |
| Relative risk of infection for 2 to 5-year-olds | 0.97 |  |
| Probability of symptomatic primary infection | 0.11 |  |
| Relative infectiousness of chronic carriers (rC) | Beta(8.34,36.08) |  |
| **Very High** | | |
| R0 Equation (dependent on rC) | 2.484 + 45.756rC + -20.057rC^2 |  |
| Relative risk of infection for 0 to 2-year-olds | 0.25 |  |
| Relative risk of infection for 2 to 5-year-olds | 1.00 |  |
| Probability of symptomatic primary infection | 0.61 |  |
| Relative infectiousness of chronic carriers (rC) | Beta(8.34,36.08) |  |

**Figure E.2: Model fit to the incidence data for the Yale compartmental model**

To calibrate the model to the vaccine effects observed in the Bangladesh cRCT, we simulated the trial, implementing a vaccine campaign for individuals aged 9 months to 15 years in the high incidence setting. We ran this simulation 1000 times, randomly sampling a value from [0,1] for the relative infectiousness of chronic carriers (rC). We then isolated the sampled rC values that led to simulations where the estimated vaccine effects were within the cRCT’s confidence intervals for overall, total, and indirect protection. The samples were used to make a beta distribution Beta(8.34,36.08) for rC that was sampled from for each epidemiological simulation. This model also used 1000 sampled estimates generated by the statistical model for the VE and waning parameters. Vaccine protection was modeled as an exponential decay function for slow waning and a gamma decay function (shape = 2) for fast waning, randomly sampling from the posterior distribution across the epidemiological simulations.

# References

1. WHO. Modelled Evidence To Support A SAGE WG On Typhoid Conjugate Vaccine (TCV) [Internet]. 2024 [cited 2026 Feb 12]. Available from: <https://www.ungm.org/Public/Notice/241135>
2. Patel PD, Patel P, Liang Y, Meiring JE, Misiri T, Mwakiseghile F, et al. Safety and efficacy of a typhoid conjugate vaccine in Malawian children. New England Journal of Medicine. 2021 Sept 16;385(12):1104–15. doi:10.1056/nejmoa2035916
3. Patel PD, Liang Y, Meiring JE, Chasweka N, Patel P, Misiri T, et al. Efficacy of typhoid conjugate vaccine: Final analysis of a 4-year, phase 3, randomised controlled trial in malawian children. The Lancet. 2024 Feb;403(10425):459–68. doi:10.1016/s0140-6736(23)02031-7
4. Qadri F, Khanam F, Liu X, Theiss-Nyland K, Biswas PK, Bhuiyan AI, et al. Protection by vaccination of children against typhoid fever with a VI-tetanus toxoid conjugate vaccine in urban Bangladesh: A cluster-randomised trial. The Lancet. 2021 Aug;398(10301):675–84. doi:10.1016/s0140-6736(21)01124-7
5. Qadri F, Khanam F, Zhang Y, Biswas PK, Voysey M, Mujadidi YF, et al. 5-year vaccine protection following a single dose of vi-tetanus toxoid conjugate vaccine in Bangladeshi children (Tyvoid): A cluster randomised trial. The Lancet. 2024 Oct;404(10461):1419–29. doi:10.1016/s0140-6736(24)01494-6
6. Gauld JS, Hu H, Klein DJ, Levine MM. Typhoid fever in Santiago, chile: Insights from a mathematical model utilizing venerable archived data from a successful Disease Control Program. PLOS Neglected Tropical Diseases. 2018 Sept 6;12(9). doi:10.1371/journal.pntd.0006759
