# Supplementary material for: Evaluating the impact and cost-effectiveness of typhoid conjugate vaccine schedules across diverse settings: A multi-model comparison
Source: PLoS Med. 2026 Oct 13;23(10):e1005280. doi: 10.1371/journal.pmed.1005280 (PMC13686463; doi:10.1371/journal.pmed.1005280)
Supplement: Supplementary file 9 [file pmed.1005280.s008.docx]

***S6 Supplementary Text: Cost-Effectiveness Analysis***

**Evaluating the Impact and Cost-Effectiveness of Typhoid Conjugate Vaccine Schedules Across Diverse Settings: A Multi-Model Comparison**

*Authors:*

Catherine G.C. Wenger^1,&^, Kyra H. Grantz^2,&^, Tigist F. Menkir^3,&^, Alina M. Muellenmeister^4,&^, Zeaan Pithawala^1^, Raymond Hutubessy^5^, Vittal Mogasale^5^, Alicia N.M. Kraay^2^, Nick Scott^4^, Romesh G. Abeysuriya^4,$^, Jason R. Andrews^3,$^, Jillian Gauld^2,$^, Nathan C. Lo^3,$^, Virginia E. Pitzer^1,$^

*Affiliations:*

1 Department of Epidemiology of Microbial Diseases, Yale School of Public Health, Yale University, New Haven, Connecticut, United States of America

2 Institute for Disease Modeling, Gates Foundation, Seattle, Washington, United States of America

3 Division of Infectious Diseases and Geographic Medicine, Department of Medicine, Stanford University, Stanford, California, United States of America

4 Burnet Institute, Melbourne, Victoria, Australia

5 Department of Performance, Finance and Delivery, World Health Organization, Geneva, Switzerland

^&^co-first authors

^$^co-last authors

Disclaimer: RH and VM are staff members of the World Health Organization. The views expressed are their personal ones and do not necessarily represent the views of their organization. KHG, AK, and JG are employees of the Gates Foundation; however, this study does not necessarily represent the views of the Gates Foundation.

Table of Contents

[Cost-Effectiveness Analysis	3](#_Toc241485698)

[A. Scenarios	3](#_Toc241485699)

[B. Cost-effectiveness model structure	4](#_Toc241485700)

[C. Parameters	6](#_Toc241485701)

[C.1 Healthcare-seeking probabilities	8](#_Toc241485702)

[C.2 Severity parameters	8](#_Toc241485703)

[C.3 Case fatality risk (CFR)	8](#_Toc241485704)

[C.4 Disability weights	9](#_Toc241485705)

[C.5 Treatment costs	9](#_Toc241485706)

[C.6 Vaccine costs	9](#_Toc241485707)

[D. Net-benefit framework	9](#_Toc241485708)

[References	11](#_Toc241485709)

# Cost-Effectiveness Analysis

## A. Scenarios

**Figure A.1: Scenario combinations for regional cost-effectiveness parameters, incidence setting, and waning of vaccine effectiveness assumptions.** Each set of scenarios was evaluated for a total of 12 analyses. CFR indicates case fatality risk.

## B. Cost-effectiveness model structure

**Figure B.1: Probability tree for typhoid treatment and outcomes.** Each branch indicates a unique treatment pathway that individuals in the model may experience. Green boxes indicate states that are associated with age-stratified treatment costs.

Yearly symptomatic incidence of typhoid fever from the epidemiological simulations of each model were used as inputs for our cost-effectiveness model simulations. Of those with symptoms, a fraction sought care and received either outpatient or inpatient treatment. Costs are age-stratified for outpatient and inpatient treatment; pediatric costs apply to individuals aged 15 years or younger, and adult costs apply to individuals aged 16 years or older. We model inpatient complications (e.g., due to ileal perforation) separately from inpatient treatment with no complications. The costs are the same due to a lack of data on the relative cost of complications compared to no complications, but separate disability weights were applied.

We planned to incorporate a distinct treatment pathway for antimicrobial-resistant (AMR) cases in our model structure; however, there was insufficient data to fully isolate costs and outcome probabilities associated with AMR. Therefore, we assumed that the treatment costs and probabilities and case fatality risk (CFRs) estimates we used encompass both resistant and sensitive cases, as the source studies we identified were primarily conducted in locations with a high prevalence of multidrug-resistant or fluoroquinolone non-susceptible cases.

Each modeling group built a cost-effectiveness model following the same structure and sampled from harmonized uncertainty distributions for each stochastic parameter (informed by estimates from the literature as described below). Three of the modeling groups (Burnet, Stanford, and Yale) used the output from 1,000 epidemiological simulations and ran one cost-effectiveness simulation for each epidemiological simulation, resulting in a total of 1,000 economic simulations. IDM ran 250 economic simulations for each of 250 epidemiological simulations for a total of 62,500 economic simulations.

## C. Parameters

**Table C.1: Cost-effectiveness parameters**

| **Parameters** | **Africa** | **Asia** | | **Distribution** | | **Source** | |
| --- | --- | --- | --- | --- | --- | --- | --- |
| ***Healthcare-seeking parameters*** | | | | | | | |
| Probability of seeking care | 0.58 (95% CI [0.42, 0.77]) | | | Beta | | [1] | |
| Probability of inpatient treatment | 0.24 (95% CI [0.08, 0.53]) | 0.10 (95% CI [0.03, 0.28]) | | Beta | | [2,3] | |
| Probability of outpatient treatment | 1 – Probability of inpatient treatment | | | Beta | | [2,3] | |
| Probability of complications | 0.32 (95% CI [0.20, 0.47]) | 0.02 (95% CI [0.001, 0.36]) | | Beta | | [2,3] | |
| ***Severity parameters*** | | | | | | | |
| Duration of severe illness | 20.0 days (IQR [14.0 - 44.0 days]) | | | Gamma | | [4] | |
| Duration of moderate illness | 14.0 days (IQR [7.0 - 25.0 days]) | | | Gamma | | [4] | |
| Duration of untreated illness | 14.0 days (IQR [8.0 - 25.0 days]) | | | Gamma | | [4] | |
| Case fatality risk (CFR) | 0.81%  (95% CI [0.10%, 6.22%]) | 0.16%  (95% CI [0.07%-0.40%]) | | Beta | | [5] | |
| ***Disability-adjusted life year (DALY) weights*** | | | | | | | |
| Disability weight, severe with complications | 0.324 (95% CI [0.220,0.442]) | | | Beta | | [6] | |
| Disability weight, severe | 0.133 (95% CI [0.0885, 0.190]) | | | Beta | | [6] | |
| Disability weight, moderate | 0.0506 (95% CI [0.0324, 0.0741]) | | | Beta | | [6] | |
| Disability weight, untreated | 0.0506 (95% CI [0.0324, 0.0741]) | | | Beta | | [6] | |
| Life expectancy | 64.0 years | 74.8 years | Fixed | | [7] | |  |
| ***Treatment costs (USD 2025)*** | | | | | | | |
| Inpatient pediatric costs | $285.01  (95% CI [$199.29, $370.74]) | $214.45  (IQR [$153.75, $326.76]) | | Log Normal | | [4,8,9] | |
| Outpatient pediatric costs | $50.41  (95% CI [$41.16, $59.68]) | $42.62  (IQR [$26.34, $72.01]) | | Log Normal | | [4,8,9] | |
| Inpatient adult costs | $445.65  (95% CI [$295.27, $596.02]) | $214.45  (IQR [$153.75, $326.76]) | | Log Normal | | [4,8,9] | |
| Outpatient adult costs | $46.77  (95% CI [$35.18, $58.36]) | $42.62  (IQR [$26.34, $72.01]) | | Log Normal | | [4,8,9] | |
| ***Vaccination costs (USD 2025)*** | | | | | | | |
| Vaccine procurement costs (i.e. vaccine price) | $1.50 | | | Fixed | | [10] | |
| Syringes & safety equipment costs (unit price) | $0.29 (95% CI [$0.28, $0.31]) | | | Log Normal | | [11] | |
| Delivery costs, routine | $2.34  (95% CI [$1.96, $2.70]) | $1.54  (95% CI [$0.94, $2.14]) | | Log Normal | | [1] | |
| Delivery costs, school-based | $1.42 (95% CI [$0.71, $2.14]) | | | Log Normal | | [12] | |
| Delivery costs, catch-up campaign | $0.50  (95% CI [$0.44, $0.56]) | $0.54  (95% CI [$0.46, $0.61]) | | Log Normal | | [1] | |

CI = Confidence interval

IQR = Interquartile range [25%, 75%]

USD = United States Dollars

### C.1 Healthcare-seeking probabilities

The probability that an individual with typhoid seeks care was based on an estimate of the relative incidence for passive versus active surveillance, as assumed by Bilcke et al. [1]. This serves as a proxy for health-seeking behavior, since passive surveillance studies capture only cases that seek care, whereas active surveillance studies capture people with typhoid who do and do not seek care.

### C.2 Severity parameters

We assumed the probability that typhoid patients receive inpatient treatment is equivalent to the proportion of severe cases of typhoid among symptomatic infections, and the complement is the probability that patients receive outpatient treatment. These probabilities, as well as the probability of complications, are based on the Severe Typhoid in Africa programme (SETA, 2016-2020), the Surveillance for Enteric Fever in Asia Project (SEAP, 2016-2019), the Strategic Typhoid Alliance across Africa and Asia (STRATAA, 2016-2018), and the National Surveillance System for Enteric Fever in India (NSSEFI) [1,2,13,14]. Specifically, for each of the two regions, we fit a beta-binomial model with random effects for study site to reported hospitalization counts among each of the study populations, using the method of moments to link the posterior of the binomial fit to a corresponding beta distribution.

Due to a lack of regional data for Africa, duration of illness parameters were generalized to both regions based on estimates from Nepal collected during the second phase of the SEAP project (SEAP II) [4]. We used the reported days unable to work for patients who did not report inpatient expenses as the duration of moderate illness due to typhoid. For the duration of severe illness, we used the same metric for patients who reported inpatient expenses. Finally, the duration of illness for untreated patients was assumed to be the days unable to work for both of these groups combined, because we assume all sequelae can occur in untreated illness. These estimates are consistent with the duration of illness used in the 2017 Global Burden of Disease (GBD) study on typhoid, which reported years lived with disability (YLDs) for typhoid [15].

### C.3 Case fatality risk (CFR)

We calculated CFRs for each region based on data from a systematic review and meta-analysis on complications and mortality of typhoid by Murthy et al. [5]. We restricted the studies to those with a median study year of 2010 or later, since the CFR for typhoid has declined since the 1960s due to improved treatment and sanitation [16]. We ran a mixed-effects meta-analysis of proportions, with the CFR as the outcome of interest and study-level random effects, separately for each region. Data on CFRs stratified by sequelae or treatment type were incomplete, so the overall CFR for each region was used uniformly in all treatment pathways.

### C.4 Disability weights

Disability weights were sourced from the GBD 2021 study’s global estimates for typhoid and were assumed to be the same for both Africa and Asia [6]. We assumed the disability weight for moderate and untreated typhoid was equivalent to the weight for acute typhoid infection. Similarly, we used the weight for intestinal perforation due to typhoid for calculating disability-adjusted life years (DALYs) for severe typhoid cases with complications. The regional life expectancy, sourced from the same data as our demographic calibration parameters, was greater for Asia than for Africa [7].

### C.5 Treatment costs

Cost estimates for Africa came from a costing cohort study conducted in Malawi that provided direct medical costs of inpatient and outpatient treatment for both children and adults [9]. The cost estimates for Asia were based on data from SEAP II, specifically cost-of-illness studies conducted in Nepal and Bangladesh [4,8]. We assumed that the direct medical costs for patients who did not report any inpatient care expenses were equivalent to outpatient costs. For patients who reported inpatient care expenses, we attributed all of their direct medical costs to inpatient treatment. There was no clear breakdown between pediatric and adult treatment costs, so we used the overall estimates for both age groups. All treatment cost parameters were adjusted for inflation and presented in 2025 US dollars.

### C.6 Vaccine costs

We do not assume Gavi support in this analysis, so the vaccine procurement cost is the UNICEF-listed cost for a middle-income country when procuring TCV doses from Biologics E or Bharat Biologics in India [10]. Syringe and safety equipment costs increase the total cost per dose to about $1.79 and came from a costing study of vaccination in 94 low- and middle-income countries [11]. Strategy-specific delivery costs came from estimates used in other TCV cost-effectiveness studies [1,12]. All vaccine cost parameters were adjusted for inflation and presented in 2025 US dollars.

## D. Net-benefit framework

The primary cost-effectiveness analysis was conducted using the net-benefit framework. This method incorporates a probabilistic sensitivity analysis of the interventions of interest to identify the uncertainty in the predicted optimal strategy. We calculated the net monetary benefit (NMB) of each strategy using the equation:

$$NMB=p*\Delta E- \Delta C$$

where *p* is the willingness-to-pay threshold, $\Delta E$ is the DALYs averted compared to no vaccination, and $\Delta C$ is the incremental cost compared to no vaccination. For each stochastic simulation and vaccination strategy, we varied the WTP threshold from $0 to $2,500. Then, the NMB for each strategy was compared across simulations to calculate the probability that a given strategy had the highest NMB at a given WTP threshold. This was then plotted for each strategy as the cost-effectiveness acceptability curve (CEAC). The cost-effectiveness acceptability frontiers (CEAFs) were derived from the CEACs to highlight only the strategy that yielded the greatest average NMB at each WTP threshold. This approach indicates the optimal strategy across a range of WTP values.

# References

1. Bilcke J, Antillón M, Pieters Z, Kuylen E, Abboud L, Neuzil KM, et al. Cost-effectiveness of routine and campaign use of typhoid VI-conjugate vaccine in Gavi-eligible countries: A modelling study. The Lancet Infectious Diseases. 2019 Jul;19(7):728–39. doi:10.1016/s1473-3099(18)30804-1
2. Marks F, Im J, Park SE, Pak GD, Jeon HJ, Wandji Nana LR, et al. Incidence of typhoid fever in Burkina Faso, Democratic Republic of the Congo, Ethiopia, Ghana, Madagascar, and Nigeria (the severe typhoid in Africa programme): A population-based study. The Lancet Global Health. 2024 Apr;12(4). doi:10.1016/s2214-109x(24)00007-x
3. Longley AT, Hemlock C, Date K, Luby SP, Andrews JR, Saha SK, et al. Illness severity and outcomes among enteric fever cases from Bangladesh, Nepal, and Pakistan: Data from the surveillance for Enteric Fever in Asia Project, 2016–2019. Clinical Infectious Diseases. 2020 Nov 1;71(Supplement_3). doi:10.1093/cid/ciaa1320
4. Mejia N, Abimbola T, Andrews JR, Vaidya K, Tamrakar D, Pradhan S, et al. Typhoid and paratyphoid cost of illness in Nepal: Patient and health facility costs from the surveillance for Enteric Fever in Asia Project II. Clinical Infectious Diseases. 2020 Nov 1;71(Supplement_3). doi:10.1093/cid/ciaa1335
5. Murthy S, Hagedoorn NN, Faigan S, Rathan MD, Marchello CS, Crump JA. Complications and mortality of typhoid fever: An updated global systematic review and meta-analysis. The Lancet Infectious Diseases. 2025 Nov; doi:10.1016/s1473-3099(25)00551-1
6. Global Burden of Disease Collaborative Network. Global Burden of Disease Study 2021 (GBD 2021) Disability Weights. Institute for Health Metrics and Evaluation (IHME). 2024; doi:https://doi.org/10.6069/485b-dx41
7. World Population Prospects 2024 | Population Division [Internet]. United Nations; 2024 [cited 2025 Dec 11]. Available from: https://www.un.org/development/desa/pd/world-population-prospects-2024
8. Mejia N, Pallas SW, Saha S, Udin J, Sayeed KMI, Garrett DO, et al. Typhoid and paratyphoid cost of illness in Bangladesh: patient and health facility costs from the Surveillance for Enteric Fever in Asia Project II. *Clin Infect Dis*. 2020 Nov 1;71(Suppl 3):S293–S305. doi: 10.1093/cid/ciaa1334.
9. Limani F, Smith C, Wachepa R, Chafuwa H, Meiring J, Noah P, et al. Estimating the economic burden of typhoid in children and adults in Blantyre, Malawi: A costing cohort study. PLOS ONE. 2022 Nov 23;17(11). doi:10.1371/journal.pone.0277419
10. UNICEF Supply Division [Internet]. 2024 [cited 2025 Dec 12]. Available from: https://www.unicef.org/supply/documents/typhoid-conjugate-vaccine-tcv-price-data
11. Portnoy A, Ozawa S, Grewal S, Norman BA, Rajgopal J, Gorham KM, et al. Costs of vaccine programs across 94 low- and middle-income countries. Vaccine. 2015 May;33. doi:10.1016/j.vaccine.2014.12.037
12. Weyant C, Hooda Y, Munira SJ, Lo NC, Ryckman T, Tanmoy AM, et al. Cost-effectiveness and public health impact of typhoid conjugate vaccine introduction strategies in Bangladesh. Vaccine. 2024 Apr;42(11):2867–76. doi:10.1016/j.vaccine.2024.03.035
13. John J, Bavdekar A, Rongsen-Chandola T, Dutta S, Gupta M, Kanungo S, et al. Burden of typhoid and paratyphoid fever in India. New England Journal of Medicine. 2023 Apr 20;388(16):1491–500. doi:10.1056/nejmoa2209449
14. Darton TC, Meiring JE, Tonks S, Khan MA, Khanam F, Shakya M, et al. The STRATAA study protocol: A programme to assess the burden of enteric fever in Bangladesh, Malawi and Nepal using prospective population census, passive surveillance, serological studies and healthcare utilisation surveys. BMJ Open. 2017 Jun;7(6). doi:10.1136/bmjopen-2017-016283
15. Stanaway JD, Reiner RC, Blacker BF, Goldberg EM, Khalil IA, Troeger CE, et al. The global burden of typhoid and paratyphoid fevers: A systematic analysis for the global burden of disease study 2017. The Lancet Infectious Diseases. 2019 Apr;19(4):369–81. doi:10.1016/s1473-3099(18)30685-6
16. Mock C, Visser L, Denno D, Maier R. Aggressive fluid resuscitation and broad spectrum antibiotics decrease mortality from typhoid ileal perforation. Tropical Doctor. 1995 Jul;25(3):115–7. doi:10.1177/004947559502500309
