# Supplementary material for: Evaluating the impact and cost-effectiveness of typhoid conjugate vaccine schedules across diverse settings: A multi-model comparison
Source: PLoS Med. 2026 Oct 13;23(10):e1005280. doi: 10.1371/journal.pmed.1005280 (PMC13686463; doi:10.1371/journal.pmed.1005280)
Supplement: Supplementary file 10 [file pmed.1005280.s009.docx]

***S7 Supplementary Text: Supplemental Results***

**Evaluating the Impact and Cost-Effectiveness of Typhoid Conjugate Vaccine Schedules Across Diverse Settings: A Multi-Model Comparison**

*Authors:*

Catherine G.C. Wenger^1,&^, Kyra H. Grantz^2,&^, Tigist F. Menkir^3,&^, Alina M. Muellenmeister^4,&^, Zeaan Pithawala^1^, Raymond Hutubessy^5^, Vittal Mogasale^5^, Alicia N.M. Kraay^2^, Nick Scott^4^, Romesh G. Abeysuriya^4,$^, Jason R. Andrews^3,$^, Jillian Gauld^2,$^, Nathan C. Lo^3,$^, Virginia E. Pitzer^1,$^

*Affiliations:*

1 Department of Epidemiology of Microbial Diseases, Yale School of Public Health, Yale University, New Haven, Connecticut, United States of America

2 Institute for Disease Modeling, Gates Foundation, Seattle, Washington, United States of America

3 Division of Infectious Diseases and Geographic Medicine, Department of Medicine, Stanford University, Stanford, California, United States of America

4 Burnet Institute, Melbourne, Victoria, Australia

5 Department of Performance, Finance and Delivery, World Health Organization, Geneva, Switzerland

^&^co-first authors

^$^co-last authors

Disclaimer: RH and VM are staff members of the World Health Organization. The views expressed are their personal ones and do not necessarily represent the views of their organization. KHG, AK, and JG are employees of the Gates Foundation; however, this study does not necessarily represent the views of the Gates Foundation.

Table of Contents

[Supplemental Results	3](#_Toc241485978)

[A. Model-predicted vaccine impact over time	3](#_Toc241485979)

[B. Vaccine impact tables	6](#_Toc241485980)

[C. Cost-effectiveness acceptability curves	14](#_Toc241485981)

[D Cost-effectiveness acceptability frontiers for the 20-year time horizon	20](#_Toc241485982)

[E. Scenario analysis: youngest age of vaccination at 15 months	24](#_Toc241485983)

[F. Scenario analysis: vaccination without catch-up campaigns	28](#_Toc241485984)

[G. Threshold analysis	32](#_Toc241485985)

[H. Health savings compared to the cost of booster doses	37](#_Toc241485986)

[I. One-way sensitivity analysis	41](#_Toc241485987)

# Supplemental Results

## A. Model-predicted vaccine impact over time

**Figure A.1 Incidence of symptomatic typhoid for booster dose strategies in the medium incidence setting for all models.**

y = year, mo = month

**Figure A.2 Incidence of symptomatic typhoid for booster dose strategies in the high incidence setting for all models.**

y = year, mo = month

**Figure A.3 Incidence of symptomatic typhoid for booster dose strategies in the very high incidence setting for all models.**

y = year, mo = month

**Figure A.4 Incidence curves of symptomatic typhoid for routine strategies in the medium incidence setting for all models.**

yrs = years, mo = month

**Figure A.5 Incidence curves of symptomatic typhoid for routine strategies in the high incidence setting for all models.**

yrs = years, mo = month

**Figure A.6 Incidence curves of symptomatic typhoid for routine strategies in the very high incidence setting for all models.**

yrs = years, mo = month

## B. Vaccine impact tables

**Figure B.1 Median cumulative proportion ever infected by age group across models and incidence settings.** The plotted values are the cumulative median proportion of individuals ever infected in each age group and 95% prediction interval (PI) across simulations. The Stanford results do not account for previously infected susceptible individuals, leading to an underestimation of the proportions (although this is likely small given the long duration of natural immunity estimated for this model). yr = year, mo = month.

**Table B.1 Doses administered per 100,000 people for each vaccination strategy and model over a 10-year time horizon.** Data is presented as the median value from all simulations of that model and a 95% prediction interval.

**Table B.2 Vaccine impact results for each vaccination strategy and model in the medium incidence, higher-case fatality risk/higher-cost African scenario over a 10-year time horizon.** Data is presented as the median value from all simulations of that model and a 95% prediction interval. Deaths are rounded to the nearest tenth, and all other values are rounded to the nearest whole number.

**Table B.3 Vaccine impact results for each vaccination strategy and model in the high incidence, higher-case fatality risk/higher-cost Africa scenario over a 10-year time horizon.** Data is presented as the median value from all simulations of that model and a 95% prediction interval. Deaths are rounded to the nearest tenth, and all other values are rounded to the nearest whole number.

**Table B.4 Vaccine impact results for each vaccination strategy and model in the very high incidence, higher-case fatality risk/higher-cost Africa scenario over a 10-year time horizon.** Data is presented as the median value from all simulations of that model and a 95% prediction interval. Deaths are rounded to the nearest tenth, and all other values are rounded to the nearest whole number.

**Table B.5 Vaccine impact results for each vaccination strategy and model in the medium incidence, lower-case fatality risk/lower-cost Asia scenario over a 10-year time horizon.** Data is presented as the median value from all simulations of that model and a 95% prediction interval. Deaths are rounded to the nearest tenth, and all other values are rounded to the nearest whole number.

**Table B.6 Vaccine impact results for each vaccination strategy and model in the high incidence, lower-case fatality risk/lower-cost Asia scenario over a 10-year time horizon.** Data is presented as the median value from all simulations of that model and a 95% prediction interval. Deaths are rounded to the nearest tenth, and all other values are rounded to the nearest whole number.

**Table B.7 Vaccine impact results for each vaccination strategy and model in the very high incidence, lower-case fatality risk/lower-cost Asia scenario over a 10-year time horizon.** Data is presented as the median value from all simulations of that model and a 95% prediction interval. Deaths are rounded to the nearest tenth, and all other values are rounded to the nearest whole number.

## C. Cost-effectiveness acceptability curves

**Figure C.1 Cost-effectiveness acceptability curves (CEACs) for the slow-waning, higher-case fatality risk/higher-cost Africa scenario for all incidence settings over a 10-year time horizon.** The lines indicate the probability that each strategy has the highest net-benefit compared to the other strategies across a range of willingness-to-pay thresholds per disability-adjusted life-year (DALY) averted by the intervention. y = year, m = month.

**Figure C.2 Cost-effectiveness acceptability curves (CEACs) for the fast-waning, higher-case fatality risk/higher-cost Africa scenario for all incidence settings over a 10-year time horizon.** The lines indicate the probability that each strategy has the highest net-benefit compared to the other strategies across a range of willingness-to-pay thresholds per disability-adjusted life-year (DALY) averted by the intervention. y = year, m = month.

**Figure C.3 Cost-effectiveness acceptability curves (CEACs) for the slow-waning, lower-case fatality risk/lower-cost Asia scenario for all incidence settings over a 10-year time horizon.** The lines indicate the probability that each strategy has the highest net-benefit compared to the other strategies across a range of willingness-to-pay thresholds per disability-adjusted life-year (DALY) averted by the intervention. y = year, m = month.

**Figure C.4 Cost-effectiveness acceptability curves (CEACs) for the fast-waning, lower-case fatality risk/lower-cost Asia scenario for all incidence settings over a 10-year time horizon.** The lines indicate the probability that each strategy has the highest net-benefit compared to the other strategies across a range of willingness-to-pay thresholds per disability-adjusted life-year (DALY) averted by the intervention. y = year, m = month.

## D Cost-effectiveness acceptability frontiers for the 20-year time horizon

**Figure D.1 Cost-effectiveness acceptability frontier (CEAF) heatmap for the slow-waning, higher-case fatality risk/higher-cost Africa scenario for all incidence settings over a 20-year time horizon.** The optimal strategy predicted by each model is indicated by the color plotted at a given willingness-to-pay threshold per disability-adjusted life-year averted by the intervention. The shading indicates the probability that the strategy will provide the highest net benefit, with darker shades representing a higher probability. This analysis includes a two-booster strategy with routine vaccination at 9 months and boosters administered at 5 and 10 years of age. y=year, m=month.

**Figure D.2 Cost-effectiveness acceptability frontier (CEAF) heatmap for the fast-waning, higher-case fatality risk/higher-cost Africa scenario for all incidence settings over a 20-year time horizon.** The optimal strategy predicted by each model is indicated by the color plotted at a given willingness-to-pay threshold per disability-adjusted life-year averted by the intervention. The shading indicates the probability that the strategy will provide the highest net benefit, with darker shades representing a higher probability. This analysis includes a two-booster strategy with routine vaccination at 9 months and boosters administered at 5 and 10 years of age. y=year, m=month.

**Figure D.3 Cost-effectiveness acceptability frontier (CEAF) heatmap for the slow-waning, lower-case fatality risk/lower-cost Asia scenario for all incidence settings over a 20-year time horizon.** The optimal strategy predicted by each model is indicated by the color plotted at a given willingness-to-pay threshold per disability-adjusted life-year averted by the intervention. The shading indicates the probability that the strategy will provide the highest net benefit, with darker shades representing a higher probability. This analysis includes a two-booster strategy with routine vaccination at 9 months and boosters administered at 5 and 10 years of age. y=year, m=month.

**Figure D.4 Cost-effectiveness acceptability frontier (CEAF) heatmap for the fast-waning, lower-case fatality risk/lower-cost Asia scenario for all incidence settings over a 20-year time horizon.** The optimal strategy predicted by each model is indicated by the color plotted at a given willingness-to-pay threshold per disability-adjusted life-year averted by the intervention. The shading indicates the probability that the strategy will provide the highest net benefit, with darker shades representing a higher probability. This analysis includes a two-booster strategy with routine vaccination at 9 months and boosters administered at 5 and 10 years of age. y=year, m=month.

## E. Scenario analysis: youngest age of vaccination at 15 months

**Figure E.1 Cost-effectiveness acceptability frontier (CEAF) heatmap considering the youngest age of vaccination at 15 months for the slow-waning, higher-case fatality risk/higher-cost Africa scenario for all incidence settings over a 10-year time horizon.** The optimal strategy predicted by each model is indicated by the color plotted at a given willingness-to-pay threshold per disability-adjusted life-year averted by the intervention. The shading indicates the probability that the strategy will provide the highest net benefit, with darker shades representing a higher probability. y=year, m=month.

**Figure E.2 Cost-effectiveness acceptability frontier (CEAF) heatmap considering the youngest age of vaccination at 15 months for the fast-waning, higher-case fatality risk/higher-cost Africa scenario for all incidence settings over a 10-year time horizon.** The optimal strategy predicted by each model is indicated by the color plotted at a given willingness-to-pay threshold per disability-adjusted life-year averted by the intervention. The shading indicates the probability that the strategy will provide the highest net benefit, with darker shades representing a higher probability. y=year, m=month.

**Figure E.3 Cost-effectiveness acceptability frontier (CEAF) heatmap considering the youngest age of vaccination at 15 months for the slow-waning, lower-case fatality risk/lower-cost Asia scenario for all incidence settings over a 10-year time horizon.** The optimal strategy predicted by each model is indicated by the color plotted at a given willingness-to-pay threshold per disability-adjusted life-year averted by the intervention. The shading indicates the probability that the strategy will provide the highest net benefit, with darker shades representing a higher probability. y=year, m=month.

**Figure E.4 Cost-effectiveness acceptability frontier (CEAF) heatmap considering the youngest age of vaccination at 15 months for the fast-waning, lower-case fatality risk/lower-cost Asia scenario for all incidence settings over a 10-year time horizon.** The optimal strategy predicted by each model is indicated by the color plotted at a given willingness-to-pay threshold per disability-adjusted life-year averted by the intervention. The shading indicates the probability that the strategy will provide the highest net benefit, with darker shades representing a higher probability. y=year, m=month.

## F. Scenario analysis: vaccination without catch-up campaigns

**Figure F.1 Cost-effectiveness acceptability frontier (CEAF) heatmap considering strategies without catch-up campaigns only for the slow-waning, higher-case fatality risk/higher-cost Africa scenario for all incidence settings over a 10-year time horizon.** The optimal strategy predicted by each model is indicated by the color plotted at a given willingness-to-pay threshold per disability-adjusted life-year averted by the intervention. The shading indicates the probability that the strategy will provide the highest net benefit, with darker shades representing a higher probability. y=year, m=month.

**Figure F.2 Cost-effectiveness acceptability frontier (CEAF) heatmap considering strategies without catch-up campaigns only for the fast-waning, higher-case fatality risk/higher-cost Africa scenario for all incidence settings over a 10-year time horizon.** The optimal strategy predicted by each model is indicated by the color plotted at a given willingness-to-pay threshold per disability-adjusted life-year averted by the intervention. The shading indicates the probability that the strategy will provide the highest net benefit, with darker shades representing a higher probability. y=year, m=month.

**Figure F.3 Cost-effectiveness acceptability frontier (CEAF) heatmap considering strategies without catch-up campaigns only for the slow-waning, lower-case fatality risk/lower-cost Asia scenario for all incidence settings over a 10-year time horizon.** The optimal strategy predicted by each model is indicated by the color plotted at a given willingness-to-pay threshold per disability-adjusted life-year averted by the intervention. The shading indicates the probability that the strategy will provide the highest net benefit, with darker shades representing a higher probability. y=year, m=month.

**Figure F.4 Cost-effectiveness acceptability frontier (CEAF) heatmap considering strategies without catch-up campaigns only for the fast-waning, lower-case fatality risk/lower-cost Asia scenario for all incidence settings over a 10-year time horizon.** The optimal strategy predicted by each model is indicated by the color plotted at a given willingness-to-pay threshold per disability-adjusted life-year averted by the intervention. The shading indicates the probability that the strategy will provide the highest net benefit, with darker shades representing a higher probability. y=year, m=month.

## G. Threshold analysis

**Figure G.1 Threshold analysis of the preferred vaccination strategy across different baseline incidence values with peak incidence in the 10- to 15-year age group.** The optimal strategy predicted by the model (from the cost-effectiveness acceptability frontier) is indicated by the color plotted for different baseline typhoid incidence rates (from 10 to 500 cases per 100,000 person-years) at a given willingness-to-pay per disability-adjusted life-year averted (from $0 to $5,000, x-axis). Results are plotted for a 10-year time horizon under the slow-waning scenario (top) and fast-waning scenario (bottom) from the higher-case fatality risk (CFR)/higher-cost African setting (left) and lower-CFR/lower-cost Asian setting (right). The red lines indicate the calibrated incidence used in the main analysis for medium incidence settings. These plots present the results for the Yale model; results for all models are included below. y=year, m=month.

**Figure G.2 Cost-effectiveness acceptability frontier across willingness-to-pay thresholds per disability-adjusted life years averted and incidence ranges for the slow-waning, higher-case fatality risk/higher-cost Africa setting over a 10-year time horizon.** The optimal strategy predicted by each model is indicated by the color plotted at a given willingness-to-pay threshold per disability-adjusted life-year averted by the intervention. y=year, m=month.

**Figure G.3 Cost-effectiveness acceptability frontier across willingness-to-pay thresholds per disability-adjusted life years averted and incidence ranges for the fast-waning, higher-case fatality risk/higher-cost Africa setting over a 10-year time horizon.** The optimal strategy predicted by each model is indicated by the color plotted at a given willingness-to-pay threshold per disability-adjusted life-year averted by the intervention. y=year, m=month.

**Figure G.4 Cost-effectiveness acceptability frontier across willingness-to-pay thresholds per disability-adjusted life years averted and incidence ranges for the slow-waning, lower-case fatality risk/lower-cost Asia setting over a 10-year time horizon.** The optimal strategy predicted by each model is indicated by the color plotted at a given willingness-to-pay threshold per disability-adjusted life-year averted by the intervention. y=year, m=month.

**Figure G.5 Cost-effectiveness acceptability frontier across willingness-to-pay thresholds per disability-adjusted life years averted and incidence ranges for the fast-waning, lower-case fatality risk/lower-cost Asia setting over a 10-year time horizon.** The optimal strategy predicted by each model is indicated by the color plotted at a given willingness-to-pay threshold per disability-adjusted life-year averted by the intervention. y=year, m=month.

## H. Health savings compared to the cost of booster doses

**Figure H.1 Cumulative 10-year costs of a 1-booster strategy compared to the cumulative health savings provided by the routine dose at 9 months in the slow-waning, higher-case fatality risk/higher-cost Africa scenario across burden settings and models.** yrs=year, mo=month, USD = United States Dollar.

**Figure H.2 Cumulative 10-year costs of a 1-booster strategy compared to the cumulative health savings provided by the routine dose at 9 months in the fast-waning, higher-case fatality risk/higher-cost Africa scenario across burden settings and models.** yrs=year, mo=month, USD = United States Dollar.

**Figure H.3 Cumulative 10-year costs of a 1-booster strategy compared to the cumulative health savings provided by the routine dose at 9 months in the slow-waning, lower-case fatality risk/lower-cost Asia scenario across burden settings and models.** yrs=year, mo=month, USD = United States Dollar.

**Figure H.4 Cumulative 10-year costs of a 1-booster strategy compared to the cumulative health savings provided by the routine dose at 9 months in the fast-waning, lower-case fatality risk/lower-cost Asia scenario across burden settings and models.** yrs=year, mo=month, USD = United States Dollar.

## I. One-way sensitivity analysis

**Figure I.1 One-way sensitivity analysis tornado diagram for the high incidence, slow-waning, higher-case fatality risk/higher-cost Africa scenario at a willingness-to-pay (WTP) threshold of $1,250.** This figure shows which parameters have the greatest influence on a strategy’s net monetary benefit (NMB) at the given WTP value. Parameters with a wider range of NMB have a greater influence on dictating the preferred strategy at that WTP. The colors indicate the positive or negative influence a parameter has on the NMB of a strategy. The parameter legend can be found in Table I.1.

**Figure I.2 One-way sensitivity analysis tornado diagram for the high incidence, fast-waning, higher-case fatality risk/higher-cost Africa scenario at a willingness-to-pay (WTP) threshold of $1,250.** This figure shows which parameters have the greatest influence on a strategy’s net-monetary benefit (NMB) at the given WTP value. Parameters with a wider range of NMB have a greater influence on dictating the preferred strategy at that WTP. The colors indicate the positive or negative influence a parameter has on the NMB of a strategy. The parameter legend can be found in Table I.1.

**Table I.1 One-way sensitivity analysis parameter descriptions.**
