# Supplementary material for: Evaluating the impact and cost-effectiveness of typhoid conjugate vaccine schedules across diverse settings: A multi-model comparison
Source: PLoS Med. 2026 Oct 13;23(10):e1005280. doi: 10.1371/journal.pmed.1005280 (PMC13686463; doi:10.1371/journal.pmed.1005280)
Supplement: Peer review history

# Editor report: Decision Letter 0

**DOI:** 10.1371/journal.pmed.1005280.r001

**Authors:** Helen Howard

13 Mar 2026

Dear Dr Wenger,

Thank you for submitting your manuscript entitled "Evaluating the Impact and Cost-Effectiveness of Typhoid Conjugate Vaccine Schedules Across Diverse Settings: A Multi-Model Comparison" for consideration by PLOS Medicine.

Your manuscript has now been evaluated by the PLOS Medicine editorial staff, and I am writing to let you know that we would like to send your submission out for external peer review.

However, before we can send your manuscript to reviewers, we need you to complete your submission by providing the metadata that is required for full assessment. To this end, please login to Editorial Manager where you will find the paper in the 'Submissions Needing Revisions' folder on your homepage. Please click 'Revise Submission' from the Action Links and complete all additional questions in the submission questionnaire.

For clinical studies, please upload a copy of your trial study protocol as a supporting information file. The study protocol should be the version submitted for approval to the institutional review board or ethics committee, should include any amendments to the study protocol, as well as the date of their approval by the institutional review or ethics committee. Please also detail any deviations from the study protocol in the Methods section of your manuscript. The editors will consider the protocol and study conduct prior to a final decision for external review.

Please re-submit your manuscript within two working days, i.e. by Mar 17 2026 11:59PM.

Login to Editorial Manager here: https://www.editorialmanager.com/pmedicine

Once your full submission is complete, your paper will undergo a series of checks in preparation for peer review. Once your manuscript has passed all checks it will be sent out for review.

Feel free to email us at plosmedicine@plos.org if you have any queries relating to your submission.

Kind regards,

Helen Howard

PLOS Medicine

# Peer review: Decision Letter 1

**DOI:** 10.1371/journal.pmed.1005280.r002

**Authors:** Andreia Cunha

18 May 2026

Dear Dr Wenger,

Sincere apologies for the delay in getting back to you with a decision, which was due to challenges in securing all the necessary advice. Many thanks for submitting your manuscript "Evaluating the Impact and Cost-Effectiveness of Typhoid Conjugate Vaccine Schedules Across Diverse Settings: A Multi-Model Comparison" (PMEDICINE-D-26-00808R1) to PLOS Medicine. The paper has been reviewed by subject experts and a statistician; their comments are included below and can also be accessed here: [LINK]

As you will see, the reviewers find your study of great interest to the field and raise some important questions and concerns that we must ask you to address in full. After discussing the paper with the editorial team and an academic editor with relevant expertise, I'm pleased to invite you to revise the paper in response to the reviewers' comments. We plan to send the revised paper to some or all of the original reviewers, and we cannot provide any guarantees at this stage regarding publication.

In addition to these revisions, you may need to complete some formatting changes, which you will receive in a follow up email. A member of our team will be in touch with a set of requests shortly. If you do not receive a separate email within a few days, please assume that checks have been completed, and no additional changes are required.

When you upload your revision, please include a point-by-point response that addresses all of the reviewer and editorial points, indicating the changes made in the manuscript and either an excerpt of the revised text or the location (eg: page and line number) where each change can be found. Please also be sure to check the general editorial comments at the end of this letter and include these in your point-by-point response. When you resubmit your paper, please include a clean version of the paper as the main article file and a version with changes tracked as a marked-up manuscript. It may also be helpful to check the guidelines for revised papers at http://journals.plos.org/plosmedicine/s/revising-your-manuscript for any that apply to your paper.

We ask that you submit your revision by Jun 18 2026 11:59PM. However, if this deadline is not feasible, please contact me by email, and we can discuss a suitable alternative.

Don't hesitate to contact me directly with any questions (acunha@plos.org).

Best regards,

Andreia

Andreia Cunha, PhD

Senior editor

PLOS Medicine

acunha@plos.org

-----------------------------------------------------------

Comments from the reviewers:

Reviewer #1: This study represents a significant technical contribution, harmonizing four transmission models (Burnet, IDM, Stanford, and Yale) to address critical policy questions regarding TCV waning. These findings are directly relevant to the WHO Strategic Advisory Group of Experts (SAGE), evaluating optimal schedules across varying incidence archetypes. Furthermore, the inclusion of healthcare-payer economic modeling and the focus on "fast" versus "slow" waning scenarios provide a robust framework for national decision-making.

As a member of the IVIR-AC who has reviewed the preliminary results, I find that the authors have addressed all previous comments and suggestions. Below are additional points for consideration:

1. While the manuscript includes the recommended PSA, the resulting prediction intervals remain wide, particularly regarding DALYs averted and cost-effectiveness. In Figure 3, scenarios involving slow waning with medium incidence and fast waning with high incidence show inconsistent results across models regarding the optimal age for routine vaccination (9 months vs. 2 years vs. 5 years). To assist policymakers in understanding the stability of these recommendations in contested epidemiological settings, please consider quantifying the percentage of simulations in which a 5-year routine schedule (or the recommended "optimal" schedules) outperforms a 9-month schedule (or other schedules) under fast-waning assumptions.

2. The primary analysis identifies a 9-month routine dose plus a 5-year booster as the most optimal strategy. However, the current models assume that booster doses restore efficacy to the high levels of the primary dose. Given the lack of empirical evidence regarding TCV booster performance, the manuscript should explicitly discuss the impact of this assumption on the projected long-term effectiveness.

3. The Stanford model consistently predicts lower vaccine impact in very high-incidence settings compared to the IDM or Yale models. While the SI attributes this to a high force of infection leading to natural infection before vaccine eligibility, the discussion requires a more transparent comparison of structural assumptions. Specifically, please consider comparing Stanford's "hybrid mode of protection" against the compartmental structures used by Yale and Burnet, perhaps by presenting the mean age of first infection across all four models. This would provide necessary clarity on these divergent outcomes.

Reviewer #2: The study utilized 4 different models to draw overall conclusion on the impact and cost-effectiveness of TCV. The manuscript was well written and methods were clearly described. Meaningful insights for TCV vaccination strategies were provided across various settings with different incidence, CFR and VE waning rates. My main comments are related to what protection is referred to for TCV VE, and the differences in vaccine cost across strategies.

Comments to the authors

1.Please state clear what vaccine effectiveness of TCV refers to, e.g. protection against infection, disease or severe disease?

2.Supplementary 5, VE against infection was used in the Burnet, IDM and Yale model, while VE for symptomatic disease was used in the Stanford model. There are likely differences between VE against infection and symptomatic disease. Could the authors give an overall description on how these have affected VE targets, waning and the main findings, and if any assumptions have been made?

3.Table 6.3.1, for the probability of outpatient treatment, please clarify "Compliment of P(inpatient treatment)"

4.Table 2, the vaccine cost for routine vaccination at 9 months was $115-129 thousand per 100,000 population, reduced to $77-79 thousand per 100,000 population for routine vaccination at 5 years. What is the main reason for this remarkable 30-40% difference? Would the comparison between strategies significantly impacted by this drop in vaccine cost?

5.Figure 1 shows that the trend of incidence was different for IDM, in particular with higher incidence than the no vaccine strategy after around 10 years. What is the main difference in the IDM model which explained the difference? Would the authors like to comment on this phenomenon?

Reviewer #3: This article is on evaluating the cost effectiveness of different typhoid vaccine schedules across various settings. As a statistical reviewer, there are elements of the paper which other reviewers with primary experience in health economics and/or typhoid disease characteristics may be better placed to comment on. However, in my opinion the article is generally well written with clear aims, relevant methods used and interpretation of results that are reasonable. The article also benefits from having separate modelling done using different approaches to give some degree of sensitivity checking to results. There are though some aspects of the article which I don't think are fully addressed at present:

1.With any modelling of this sort, irrespective of the choice of model the interpretation of results is heavily dependent on the assumptions that are being used. There does appear to have been substantial efforts made to address this as part of the article as far as is possible. For some assumptions though I think it is relevant to emphasise in conclusions that there is a wide range of source data estimates (eg in Africa case fatality risk 95% CI 0.10% - 6.22%) that could potentially have a non-negligible impact on results. This is further illustrated in results where as probably anticipated the CFR value has a substantial impact on the outcomes (lines 475-478, figure S7.9) but that there is a lot of uncertainty in this value from the source data.

2.In a similar manner to comment above, single values of vaccine effectiveness are given in table 1 but the referenced papers have reasonably wide confidence intervals for these values, and isn't clear whether what is used are the best/worst/mid-case scenario and how that could affect results

3.Although acknowledged as a limitation in lines 622-627 and that this may be considered as part of future analysis, it seems a substantial drawback that the vaccination is considered in isolation as to it's effectiveness/cost when there are potentially other sanitation or hygiene interventions that could instead be of equal or potentially even greater benefit than the vaccine. It may be difficult, if not impossible, to include this in models and alternative approaches are unlikely to be as relatively quick to apply as a vaccine would be. Nonetheless, I think would add substantially to the paper if more detail as to feasibility of other approaches (cost, time, cases and outcomes averted) could be outlined to place the vaccine approach in context.

4.Catch-up campaigns are mentioned throughout but as far as I can tell it isn't explicitly stated what this involves and additional costs/times that may be relevant to consider. Additionally I don't think it is clear as to whether this is being stated as applicable for all methods to imply that higher vaccination of all eligible individuals would be achievable than what is specified as being assumed in table 1 (uniform 0.5-1 from measles vaccine).

5.The models seem to assume that implementation will be possible at a fixed point in time (eg 9 months) with some catch up to complete coverage (as above comment). Although this may be all that is reasonable to include in terms of modelling, I think that some comment on the implementation of this is justified and what sort of visit window may be possible in practice as this would also affect whether single dose or booster strategies could be achievable.

6.It isn't obvious as to why 97.5% confidence intervals are specified in lines 211-212 instead of the more usual 95% level and if this implies one sided tests.

7.It is stated in lines 97-98 that the majority of disease burden is for children under 15 years old with a peak in this setting of between 2-4 year old. I don't believe though if this is what is then seen with model output in figure 1, in that the uniform distribution of non-vaccine group suggests incidence remains constant all the way between 0-20 years instead of also changing/reducing over time.

8.Some of the cells for Stanford are missing in supplementary table of model characteristics (S1_Table_model_characteristics.xlsx)

Reviewer #4: Comments

This manuscript aims to address an important question regarding the optimal schedule for typhoid vaccination given its waning protection in terms of economic and health impact using four different models. A few aspects could be refined.

Page 4, "In 2018, the World Health Organization (WHO) recommended the introduction of the typhoid conjugate vaccine (TCV) into the childhood routine immunization programs for infants and children aged 6 months to 15 years old in endemic countries, but vaccine adoption has been slow ": Can the authors clarify what the current WHO recommendation regarding TCV use or implementation strategies.

Page 4, "Vaccine efficacy data from TCV trials in children less than 15 years in Malawi and Bangladesh show differences in the duration of vaccine protection": It would be helpful if the authors elaborate on observed differences between the two settings and what their implications for model assumptions?

Page 4, "Comparing the results of multiple models can inform the robustness of model predictions and indicate which assumptions and parameters have the greatest influence on the simulated impacts" : I think a concise overview of modeling approaches would provide a context given that they are repeatedly referred in methods and results.

Page 6, "Harmonized parameters and assumptions": what do the authors mean by harmonization?

Page 16, "Figures 3 and 4 show the preferred vaccination strategies across WTP thresholds from $0 to $2,500 per DALY averted for the four models and the different incidence settings and waning scenarios [24] " : It is not clear what information is supported by reference 24. Please clarify. Consider in methods if it relates to methodological details.

Page 23, Can the authors clarify whether data from only two trials (Malawi / Bangladesh) were used or if any additional trials were considered as well, but not presented.

Any attachments provided with reviews can be seen via the following link: [LINK]

--------------------------------------------------------- ---

General editorial requests:

*We ask every co-author listed on the manuscript to fill in a contributing author statement, making sure to declare all competing interests. If any of the co-authors have not filled in the statement, we will remind them to do so when the paper is revised. If all statements are not completed in a timely fashion this could hold up the re-review process. If new competing interests are declared later in the revision process, this may also hold up the submission. Should there be a problem getting one of your co-authors to fill in a statement we will be in contact. Please do not add or remove authors without first discussing this with the handling editor. You can see our competing interests policy here: http://journals.plos.org/plosmedicine/s/competing-interests.

*Please upload any figures associated with your paper as individual TIF or EPS files with 300dpi resolution at resubmission; please read our figure guidelines for more information on our requirements: http://journals.plos.org/plosmedicine/s/figures. While revising your submission, we strongly recommend that you use PLOS's NAAS tool (https://ngplosjournals.pagemajik.ai/artanalysis) to test your figure files. NAAS can convert your figure files to the TIFF file type and meet basic requirements (such as print size, resolution), or provide you with a report on issues that do not meet our requirements and that NAAS cannot fix.

After uploading your figures to PLOS's NAAS tool - https://ngplosjournals.pagemajik.ai/artanalysis, NAAS will process the files provided and display the results in the "Uploaded Files" section of the page as the processing is complete.

If the uploaded figures meet our requirements (or NAAS is able to fix the files to meet our requirements), the figure will be marked as "fixed" above. If NAAS is unable to fix the files, a red "failed" label will appear above.

When NAAS has confirmed that the figure files meet our requirements, please download the file via the download option, and include these NAAS processed figure files when submitting your revised manuscript.

*Please ensure that the paper adheres to the PLOS Data Availability Policy (see http://journals.plos.org/plosmedicine/s/data-availability), which requires that all data underlying the study's findings be provided in a repository or as Supporting Information. For data residing with a third party, authors are required to provide instructions with contact information (web or email address) for obtaining the data. Please note that a study author cannot be the contact person for the data. PLOS journals do not allow statements supported by "data not shown" or "unpublished results." For such statements, authors must provide supporting data or cite public sources that include it.

*We expect all researchers with submissions to PLOS in which author-generated code underpins the findings in the manuscript to make all author-generated code available without restrictions upon publication of the work. In cases where code is central to the manuscript, we require the code to be made available as a condition of publication. Authors are responsible for ensuring that the code is reusable and well documented. Please make any custom code available, either as part of your data deposition or as a supplementary file. Please add a sentence to your data availability statement regarding any code used in the study, e.g. "The code used in the analysis is available from Github [URL] and archived in Zenodo [DOI link]" Please review our guidelines at https://journals.plos.org/plosmedicine/s/materials-software-and-code-sharing and ensure that your code is shared in a way that follows best practice and facilitates reproducibility and reuse. Because Github depositions can be readily changed or deleted, we encourage you to make a permanent DOI'd copy (e.g. in Zenodo) and provide the URL.

*Please ensure that the study is reported according to the most relevant guideline and include the completed corresponding checklist as Supporting Information. When completing the checklist, please use section and paragraph numbers, rather than page numbers. Please add the following statement, or similar, to the Methods: "This study is reported as per ... guideline (S1 Checklist)."

FORMATTING - GENERAL

*Abstract: Please structure your abstract using the PLOS Medicine headings (Background, Methods and Findings, Conclusions). Please combine the Methods and Findings sections into one section.

*At this stage, we ask that you include a short, non-technical Author Summary of your research to make findings accessible to a wide audience that includes both scientists and non-scientists. The Author Summary should immediately follow the Abstract in your revised manuscript. This text is subject to editorial change and should be distinct from the scientific abstract. Ideally each sub-heading should contain 2-3 single sentence, concise bullet points containing the most salient points from your study. In the final bullet point of 'What Do These Findings Mean?', please include the main limitations of the study in non-technical language. Please see our author guidelines for more information: https://journals.plos.org/plosmedicine/s/revising-your-manuscript#loc-author-summary.

*Please express the main results with 95% CIs as well as p values. When reporting p values please report as p<0.001 and where higher as the exact p value p=0.002, for example. Throughout, suggest reporting statistical information as follows to improve clarity for the reader "22% (95% CI [13%,28%]; p</=)". Please be sure to define all numerical values at first use.

*Please include page numbers and line numbers in the manuscript file. Use continuous line numbers (do not restart the numbering on each page).

*Please cite the reference numbers in square brackets. Citations should precede punctuation.

FIGURES AND TABLES

*Please provide titles and legends for all figures and tables (including those in Supporting Information files).

*Please define all abbreviations used in each figure/table (including those in Supporting Information files).

*Please consider avoiding the use of red and green in order to make your figure more accessible to those with color blindness.

SUPPLEMENTARY MATERIAL

*Please note that supplementary material will be posted as supplied by the authors. Therefore, please amend it according to the relevant comments outlined here.

*Please cite your Supporting Information as outlined here: https://journals.plos.org/plosmedicine/s/supporting-information

REFERENCES

*PLOS uses the numbered citation (citation-sequence) method and first six authors, et al.

*Please ensure that journal name abbreviations match those found in the National Center for Biotechnology Information (NCBI) databases (http://www.ncbi.nlm.nih.gov/nlmcatalog/journals), and are appropriately formatted and capitalised.

*Where website addresses are cited, please include the complete URL and specify the date of access (e.g. [accessed: 12/06/2023]).

*Please also see https://journals.plos.org/plosmedicine/s/submission-guidelines#loc-references for further details on reference formatting.

MODELLING STUDIES

The following list is derived from Geoffrey P Garnett, Simon Cousens, Timothy B Hallett, Richard Steketee, Neff Walker. Mathematical models in the evaluation of health programmes. (2011) Lancet DOI:10.1016/S0140-6736(10)61505-X:

*If pertinent, please provide a diagram that shows the model structure, including how the natural history of the disease is represented, the process and determinants of disease acquisition, and how the putative intervention could affect the system.

*Please provide a complete list of model parameters, including clear and precise descriptions of the meaning of each parameter, together with the values or ranges for each, with justification or the primary source cited and important caveats about the use of these values noted.

*Please provide a clear statement about how the model was fitted to the data, including goodness-of-fit measure, the numerical algorithm used, which parameter varied, constraints imposed on parameter values, and starting conditions.

*For uncertainty analyses, please state the sources of uncertainties quantified and not quantified [can include parameter, data, and model structure].

*Please provide sensitivity analyses to identify which parameter values are most important in the model. Uncertainty estimates seek to derive a range of credible results on the basis of an exploration of the range of reasonable parameter values. The choice of method should be presented and justified.

*Please discuss the scientific rationale for the choice of model structure and identify points where this choice could influence conclusions drawn. Please also describe the strength of the scientific basis underlying the key model assumptions.

*For studies that develop a prediction model or evaluate its performance, please ensure that the study is reported according to the TRIPOD statement (https://www.equator-network.org/reporting-guidelines/tripod-statement) and include the completed checklist as Supporting Information. Please add the following statement, or similar, to the Methods: "This study is reported as per the Transparent Reporting of a Multivariable Prediction Model for Individual Prognosis Or Diagnosis (TRIPOD) statement (S1 Checklist)." For studies using machine learning, please use the TRIPOD-AI checklist. When completing the checklist, please use section and paragraph numbers, rather than page numbers.

HEALTH ECONOMICS / COST-EFFECTIVENESS STUDIES

*Please ensure that the study is reported according to the CHEERS guideline (available from: https://www.equator-network.org/reporting-guidelines/cheers) and include the completed checklist as Supporting Information. Please add the following statement, or similar, to the Methods: "This study is reported as per the Strengthening the Consolidated Health Economic Evaluation Reporting Standards 2022 (CHEERS 2022) Statement (S1 Checklist)." When completing the checklist, please use section and paragraph numbers, rather than page numbers.

# Author comment: Author response to Decision Letter 2

**DOI:** 10.1371/journal.pmed.1005280.r003

6 Jul 2026

Attachment Submitted filename: Response_to_Reviewers.pdf

# Peer review: Decision Letter 2

**DOI:** 10.1371/journal.pmed.1005280.r004

**Authors:** Andreia Cunha

3 Sep 2026

Dear Dr. Wenger,

Sincere apologies for the delay in getting back to you with a decision and thank you very much for re-submitting your manuscript "Evaluating the Impact and Cost-Effectiveness of Typhoid Conjugate Vaccine Schedules Across Diverse Settings: A Multi-Model Comparison" (PMEDICINE-D-26-00808R2) for review by PLOS Medicine.

I have discussed the paper with my colleagues and the academic editor and it was also seen again by all the original reviewers. I am pleased to say that provided the remaining editorial and production issues are dealt with we are planning to accept the paper for publication in the journal.

The remaining issues that need to be addressed are listed at the end of this email. Any accompanying reviewer attachments can be seen via the link below. Please take these into account before resubmitting your manuscript:

[LINK]

***Please note while forming your response, if your article is accepted, you may have the opportunity to make the peer review history publicly available. The record will include editor decision letters (with reviews) and your responses to reviewer comments. If eligible, we will contact you to opt in or out.***

In revising the manuscript for further consideration here, please ensure you address the specific points made by each reviewer and the editors. In your rebuttal letter you should indicate your response to the reviewers' and editors' comments and the changes you have made in the manuscript. Please submit a clean version of the paper as the main article file. A version with changes marked must also be uploaded as a marked up manuscript file.

Please also check the guidelines for revised papers at http://journals.plos.org/plosmedicine/s/revising-your-manuscript for any that apply to your paper. If you haven't already, we ask that you provide a short, non-technical Author Summary of your research to make findings accessible to a wide audience that includes both scientists and non-scientists. The Author Summary should immediately follow the Abstract in your revised manuscript. This text is subject to editorial change and should be distinct from the scientific abstract.

In addition to these revisions, you may need to complete some formatting changes, which you will receive in a follow up email. A member of our team will be in touch with a set of requests shortly. If you do not receive a separate email within a few days, please assume that checks have been completed, and no additional changes are required.

We expect to receive your revised manuscript within 1 week. Please email us (plosmedicine@plos.org) if you have any questions or concerns.

We ask every co-author listed on the manuscript to fill in a contributing author statement. If any of the co-authors have not filled in the statement, we will remind them to do so when the paper is revised. If all statements are not completed in a timely fashion this could hold up the re-review process. Should there be a problem getting one of your co-authors to fill in a statement we will be in contact. YOU MUST NOT ADD OR REMOVE AUTHORS UNLESS YOU HAVE ALERTED THE EDITOR HANDLING THE MANUSCRIPT TO THE CHANGE AND THEY SPECIFICALLY HAVE AGREED TO IT.

Please ensure that the paper adheres to the PLOS Data Availability Policy (see http://journals.plos.org/plosmedicine/s/data-availability), which requires that all data underlying the study's findings be provided in a repository or as Supporting Information. For data residing with a third party, authors are required to provide instructions with contact information for obtaining the data. PLOS journals do not allow statements supported by "data not shown" or "unpublished results." For such statements, authors must provide supporting data or cite public sources that include it.

To enhance the reproducibility of your results, we recommend that you deposit your laboratory protocols in protocols.io, where a protocol can be assigned its own identifier (DOI) such that it can be cited independently in the future. Additionally, PLOS ONE offers an option to publish peer-reviewed clinical study protocols. Read more information on sharing protocols at https://plos.org/protocols?utm_medium=editorial-email&utm_source=authorletters&utm_campaign=protocols

Please review your reference list to ensure that it is complete and correct. If you have cited papers that have been retracted, please include the rationale for doing so in the manuscript text, or remove these references and replace them with relevant current references. Any changes to the reference list should be mentioned in the rebuttal letter that accompanies your revised manuscript.

Please note, when your manuscript is accepted, an uncorrected proof of your manuscript will be published online ahead of the final version, unless you've already opted out via the online submission form. If, for any reason, you do not want an earlier version of your manuscript published online or are unsure if you have already indicated as such, please let the journal staff know immediately at plosmedicine@plos.org.

If you have any questions in the meantime, please contact me or the journal staff on plosmedicine@plos.org.

We look forward to receiving the revised manuscript by Sep 11 2026 11:59PM.

Sincerely,

Andreia Cunha, PhD

Senior Editor

PLOS Medicine

plosmedicine.org

------------------------------------------------------------

Requests from Editors:

GENERAL EDITORIAL REQUESTS

* At this stage, we ask that you include a short, non-technical Author Summary of your research to make findings accessible to a wide audience that includes both scientists and non-scientists. The Author Summary should immediately follow the Abstract in your revised manuscript. This text is subject to editorial change and should be distinct from the scientific abstract. Ideally each sub-heading should contain 2-3 single sentence, concise bullet points containing the most salient points from your study. In the final bullet point of ‘What Do These Findings Mean?’ Please include the main limitations of the study in non-technical language.

Please see our author guidelines for more information: https://journals.plos.org/plosmedicine/s/revising-your-manuscript#loc-author-summary.

* Please confirm that your title complies with PLOS Medicine's style. Your title must be nondeclarative and not a question. It should begin with main concept if possible. "Effect of" should be used only if causality can be inferred, i.e., for an RCT. Please place the study design ("A randomized controlled trial," "A retrospective study," "A modelling study," etc.) in the subtitle (ie, after a colon).

* Please confirm that your abstract complies with our requirements, including format (three sections: Background, Methods and Findings, and Conclusions) and providing all the information relevant to this study type https://journals.plos.org/plosmedicine/s/submission-guidelines#loc-abstract

* Please ensure that the Introduction ends with a clear description of the study question or hypothesis.

* Please ensure that all abbreviations are defined at first use throughout the text.

* Please confirm that all numbers presented in the abstract are present and identical to numbers presented in the main manuscript text.

GENERAL

* Please include a statement regarding use or non-use of AI tools and technologies at the end of the Methods. Please specify the name(s) and version(s) of any tool(s) used and a description of their use, including how the AI-assisted or generated content was evaluated by authors, and a clear statement of which aspects of the study, article contents, data, or supporting files were affected or generated by AI tool usage. Please also include a statement specifying who retains responsibility for the final manuscript. See our guidelines for more information: https://journals.plos.org/plosmedicine/s/ethical-publishing-practice#loc-artificial-intelligence-tools-and-technologies.

* Please review your text for claims of novelty or primacy (e.g. 'for the first time') and remove this language. In addition, please check that any use of statistical terms (such as trend or significant) are supported by the data, and if not please remove them.

* In the last sentence of the Abstract Methods and Findings section, please describe the main limitation(s) of the study's methodology.

* In the abstract, please include the important dependent variables that are adjusted for in the analyses.

* In the author summary, in the final bullet point of 'What Do These Findings Mean?', please include the main limitations of the study in non-technical language.

FUNDING STATEMENT

* The funding statement should include: specific grant numbers, initials of authors who received each award, URLs to sponsors’ websites. Also, please state whether any sponsors or funders (other than the named authors) played any role in study design, data collection and analysis, the decision to publish, or preparation of the manuscript. If they had no role in the research, include this sentence: “The funders had no role in study design, data collection and analysis, decision to publish, or preparation of the manuscript.”

* It appears that one or more study authors is affiliated with one or more of the agencies that funded the study. Thus, the statement “The funders had no role in study design, data collection and analysis, decision to publish, or preparation of the manuscript” does not apply. Please revise the Financial Disclosure accordingly, as in "[Author name] is [author's role] at [funding agency]. The funders had no other role in study design…..”

COMPETING INTERESTS STATEMENT

* All authors must declare their relevant competing interests per the PLOS policy, which can be seen here: https://journals.plos.org/plosmedicine/s/competing-interests For authors with ties to industry, please indicate whether any of the interests has a financial stake in the results of the current study.

FIGURES

* Please provide titles and legends for all figures and tables (including those in Supporting Information files). Please define all acronyms used in each figure or table in its corresponding legend.

* Please consider avoiding the use of red and green in order to make your figure more accessible

* Please provide the unadjusted comparisons as well as the adjusted comparisons in all relevant Tables

* Please specify the variables controlled for in all relevant Tables

ACKNOWLEDGMENTS

* Please include an Acknowledgments section in your manuscript. The Acknowledgements should not include funder information.

MODELING STUDIES

For more details on the requests below please see Geoffrey P Garnett, Simon Cousens, Timothy B Hallett, Richard Steketee, Neff Walker. Mathematical models in the evaluation of health programmes. (2011) Lancet DOI:10.1016/S0140-6736(10)61505-X.

* Please provide a diagram that shows the model structure, including how the disease natural history is represented, the process and determinants of disease acquisition, and how the putative intervention could affect the system.

* Please provide a complete list of model parameters, including clear and precise descriptions of the meaning of each parameter, together with the values or ranges for each, with justification or the primary source cited, and important caveats about the use of these values noted.

* Please provide a clear statement about how the model was fitted to the data including where relevant goodness-of-fit measure, the numerical algorithm used, which parameter varied, constraints imposed on parameter values, and starting conditions.

* For uncertainty analyses, please state the sources of uncertainties quantified and not quantified this can include parameter, data, and model structure.

* Please provide sensitivity analyses to identify which parameter values are most important in the model. Uncertainty estimates seek to derive a range of credible results on the basis of an exploration of the range of reasonable parameter values. The choice of method should be presented and justified.

* Please discuss the scientific rationale for this choice of model structure and identify points where this choice could influence conclusions drawn. Please also describe the strength of the scientific basis underlying the key model assumptions.

Comments from Reviewers:

Reviewer #1: The authors have addressed all my comments.

Reviewer #2: The authors have addressed all of my comments. Thank you!

Reviewer #3: Reviewer 3

The authors have reasonably addressed all previous comments and as a result in my opinion there are no further issues to resolve.

Reviewer #4: Authors have addressed the comments adequately.

Any attachments provided with reviews can be seen via the following link:

[LINK]

# Author comment: Author response to Decision Letter 3

**DOI:** 10.1371/journal.pmed.1005280.r005

15 Sep 2026

Attachment Submitted filename: Response_to_Editor__R3.pdf

# Editor report: Decision Letter 3

**DOI:** 10.1371/journal.pmed.1005280.r006

**Authors:** Andreia Cunha

22 Sep 2026

Dear Dr Wenger,

On behalf of my colleagues and the Academic Editor, Mirjam Kretzschmar, I am pleased to inform you that we have agreed to publish your manuscript "Evaluating the Impact and Cost-Effectiveness of Typhoid Conjugate Vaccine Schedules Across Diverse Settings: A Multi-Model Comparison" (PMEDICINE-D-26-00808R3) in PLOS Medicine.

Before your manuscript can be formally accepted you will need to complete some formatting changes, which you will receive in a follow up email. Please be aware that it may take several days for you to receive this email; during this time no action is required by you. Once you have received these formatting requests, please note that your manuscript will not be scheduled for publication until you have made the required changes.

Please note that we ask you to clarify if line 48 in the Abstract is consistent with the statement in the Author Summary on lines 75-77 as line 48 indicates low CFR in high income setting and lines 75-77 suggest the opposite.

In the meantime, please log into Editorial Manager at http://www.editorialmanager.com/pmedicine/, click the "Update My Information" link at the top of the page, and update your user information to ensure an efficient production process.

PRESS

We frequently collaborate with press offices. If your institution or institutions have a press office, please notify them about your upcoming paper at this point, to enable them to help maximise its impact. If the press office is planning to promote your findings, we would be grateful if they could coordinate with medicinepress@plos.org. If you have not yet opted out of the early version process, we ask that you notify us immediately of any press plans so that we may do so on your behalf.

We also ask that you take this opportunity to read our Embargo Policy regarding the discussion, promotion and media coverage of work that is yet to be published by PLOS. As your manuscript is not yet published, it is bound by the conditions of our Embargo Policy. Please be aware that this policy is in place both to ensure that any press coverage of your article is fully substantiated and to provide a direct link between such coverage and the published work. For full details of our Embargo Policy, please visit http://www.plos.org/about/media-inquiries/embargo-policy/.

To enhance the reproducibility of your results, we recommend that you deposit your laboratory protocols in protocols.io, where a protocol can be assigned its own identifier (DOI) such that it can be cited independently in the future. Additionally, PLOS ONE offers an option to publish peer-reviewed clinical study protocols. Read more information on sharing protocols at https://plos.org/protocols?utm_medium=editorial-email&utm_source=authorletters&utm_campaign=protocols

Thank you again for submitting to PLOS Medicine. We look forward to publishing your paper.

Sincerely,

Alison Farrell, PhD

Senior Editor

PLOS Medicine
